# Allele Sorting as a Novel Approach to Resolving the Origin of Allotetraploids Using Hyb-Seq Data: A Case Study of the Balkan Mountain Endemic *Cardamine barbaraeoides*

**DOI:** 10.3389/fpls.2021.659275

**Published:** 2021-04-28

**Authors:** Marek Šlenker, Adam Kantor, Karol Marhold, Roswitha Schmickl, Terezie Mandáková, Martin A. Lysak, Marián Perný, Michaela Caboňová, Marek Slovák, Judita Zozomová-Lihová

**Affiliations:** ^1^Institute of Botany, Plant Science and Biodiversity Centre, Slovak Academy of Sciences, Bratislava, Slovakia; ^2^Department of Botany, Faculty of Science, Charles University, Prague, Czechia; ^3^Institute of Botany, The Czech Academy of Sciences, Průhonice, Czechia; ^4^Central European Institute of Technology, Masaryk University, Brno, Czechia; ^5^Department of Experimental Biology, Faculty of Science, Masaryk University, Brno, Czechia; ^6^National Centre for Biomolecular Research, Faculty of Science, Masaryk University, Brno, Czechia; ^7^Independent Researcher, Žibritov, Slovakia

**Keywords:** allopolyploidy, Balkan endemism, genomic *in situ* hybridization, Hyb-Seq, nrDNA, Pindhos Mts., read-backed phasing, target enrichment

## Abstract

Mountains of the Balkan Peninsula are significant biodiversity hotspots with great species richness and a large proportion of narrow endemics. Processes that have driven the evolution of the rich Balkan mountain flora, however, are still insufficiently explored and understood. Here we focus on a group of *Cardamine* (Brassicaceae) perennials growing in wet, mainly mountainous habitats. It comprises several Mediterranean endemics, including those restricted to the Balkan Peninsula. We used target enrichment with genome skimming (Hyb-Seq) to infer their phylogenetic relationships, and, along with genomic *in situ* hybridization (GISH), to resolve the origin of tetraploid *Cardamine barbaraeoides* endemic to the Southern Pindos Mts. (Greece). We also explored the challenges of phylogenomic analyses of polyploid species and developed a new approach of allele sorting into homeologs that allows identifying subgenomes inherited from different progenitors. We obtained a robust phylogenetic reconstruction for diploids based on 1,168 low-copy nuclear genes, which suggested both allopatric and ecological speciation events. In addition, cases of plastid–nuclear discordance, in agreement with divergent nuclear ribosomal DNA (nrDNA) copy variants in some species, indicated traces of interspecific gene flow. Our results also support biogeographic links between the Balkan and Anatolian–Caucasus regions and illustrate the contribution of the latter region to high Balkan biodiversity. An allopolyploid origin was inferred for *C. barbaraeoides*, which highlights the role of mountains in the Balkan Peninsula both as refugia and melting pots favoring species contacts and polyploid evolution in response to Pleistocene climate-induced range dynamics. Overall, our study demonstrates the importance of a thorough phylogenomic approach when studying the evolution of recently diverged species complexes affected by reticulation events at both diploid and polyploid levels. We emphasize the significance of retrieving allelic and homeologous variation from nuclear genes, as well as multiple nrDNA copy variants from genome skim data.

## Introduction

The Mediterranean Basin is one of Earth’s major biodiversity centers (Myers et al., 2000) harboring several regional hotspots with increased levels of species richness and endemism (Médail and Quézel, 1997; Thompson, 2020). Processes that have given rise to such biodiversity hotspots at a finer scale are complex and reflect interactions of climatic, geological, and biogeographic history of the Mediterranean region (Hewitt, 2011; Nieto Feliner, 2014; Thompson, 2020). Areas of high endemism are concentrated particularly on islands and in mountains, which provide favorable conditions for both speciation and long-term population persistence (Médail and Quézel, 1997; Stevanović et al., 2007; Panitsa et al., 2018; Thompson, 2020). Complex mountainous landscape has a buffering effect on climate change and enables species to survive periods of climatic fluctuations through minor range shifts (Médail and Diadema, 2009; Harrison and Noss, 2017; Muellner-Riehl et al., 2019). Mountains, however, are not just reservoirs, but also cradles of diversity. Great habitat diversity over short geographic distances and high topographic complexity of the mountains creates opportunities in which both adaptive and nonadaptive speciation may occur (Harrison and Noss, 2017; Perrigo et al., 2020). These factors also favored the evolution of narrow endemism in the Mediterranean (Thompson, 2020). In addition, range or niche shifts in response to geological and climatic events may bring vicariant taxa into contact and cause hybridization, with or without a ploidy level increase (Nieto Feliner, 2014). Although hybridization and polyploidization are recognized as significant processes for plant evolution and speciation (Soltis and Soltis, 2009; Soltis et al., 2014), their frequency and contribution to the high species diversity and endemism in the Mediterranean are still poorly understood (Marques et al., 2018; Thompson, 2020).

Here, we focus on the mainland area of the central and southernBalkan Peninsula, which is one of the regional biodiversity hotspotswith a large proportion of narrow endemics (Stevanović et al., 2007; Georghiou and Delipetrou, 2010; Tomović et al., 2014). Despite extensive botanical explorations and well-described endemism patterns in this area, speciation processes that have driven the evolution of the rich mountain flora are still not sufficiently explored. Mainly allopatric speciation often accompanied by reticulate and polyploid evolution has been suggested in recent studies (López-Vinyallonga et al., 2015; Olšavská et al., 2016; Durović et al., 2017; Španiel et al., 2017). High species diversity in this area may also be connected with adjacent Anatolia, which is recognized as a center of lineage diversification in several plant genera and a possible source for the colonization of the Balkan Peninsula (e.g., Ansell et al., 2011; Surina et al., 2014; Caković et al., 2015; Koch et al., 2017). Plant migration via two dispersal corridors, the North Anatolian Mountains or the Taurus Mountains, has been proposed, which was enhanced by land bridges that existed since the Messinian salinity crisis until the Pliocene–Pleistocene transition (Bilgin, 2011; Kaya and Çiplak, 2017; Özüdoğru and Mummenhoff, 2020).

*Cardamine* L. (Brassicaceae) is a worldwide distributed and species-rich genus (>200 spp.), which has one of its diversity centers located in the European Mediterranean (Marhold et al., 2004, 2018; Lihová and Marhold, 2006; Carlsen et al., 2009; Kučera et al., 2010). The target group of species studied here comprises approximately 30 taxa, both at species and subspecies levels, and includes a few widespread taxa distributed across Europe, several endemics confined to Southern Europe, and also some species from SW Asia (mainly the Anatolian and Caucasus regions). They have commonly been delimited as three related diploid–polyploid species complexes: the *Cardamine amara*, *Cardamine pratensis*, and *Cardamine raphanifolia* groups (Lihová et al., 2004a; Marhold et al., 2004, 2018). In contrast to this traditional, morphology-based delimitation, phylogenetic reconstructions suggested the existence of only two complexes resolved as respective monophyletic clades, one comprising the *C. amara* complex and the other the remaining species (Marhold et al., 2004; Carlsen et al., 2009). The crown group ages of both clades have been dated back to the Pliocene (approximately 3–4 Mya), and divergence of the extant species likely occurred during the Pleistocene (Huang et al., 2020). Most of the species diversity of these complexes is concentrated in Mediterranean mountains, which host several diploid and polyploid endemics (Marhold et al., 2018). Polyploid origins have been resolved or hypothesized in only a few cases (Lihová et al., 2004a, 2006; Perný et al., 2005a), and even at the diploid level, species relationships within the complexes have remained poorly understood (Lihová et al., 2004a; Marhold et al., 2004). In the Balkan Peninsula, diploid endemics prevail, and these include *Cardamine penzesii* Ančev et Marhold, *Cardamine rivularis* Schur, *C. amara* subsp. *balcanica* Ančev, Marhold et Kit Tan, and *Cardamine acris* Griseb. with three subspecies recognized. In addition, tetraploid populations from the Pindos Mts. in northwestern Greece have been reported and attributed to *Cardamine barbaraeoides* Halácsy. It is a species with an uncertain circumscription and unknown polyploid origin (Marhold et al., 2018).

High-throughput DNA sequencing has brought excellent opportunities to improve phylogenetic inferences, particularly when facing difficult evolutionary cases, such as rapid radiations or recent speciation characterized by low genetic divergence and presence of incomplete lineage sorting (ILS) often complicated by hybridization and polyploidy (Schmickl et al., 2016; Nikolov et al., 2019; Karbstein et al., 2020; Larridon et al., 2020). Disentangling reticulate and polyploid evolution, however, has been a difficult task, and phylogenomic studies on polyploids have lagged behind (Oxelman et al., 2017; Rothfels, 2021). Recent advances in this respect (see, e.g., Kamneva et al., 2017; Morales-Briones et al., 2018; Carter et al., 2019; Brandrud et al., 2020) have opened up new perspectives on analyses of polyploid species complexes. Approaches that account simultaneously for ILS and reticulation have been developed and improved (Oberprieler et al., 2017; Wen et al., 2018; Cao et al., 2019). Those network methods can provide significant insights into the evolution of polyploids based on multilocus sequence data (e.g., Kamneva et al., 2017; Morales-Briones et al., 2018). Still, standard practice when assembling sequencing reads is to generate a single consensus sequence per locus and individual, which represents a strong violation for allopolyploid genomes. The outcome of such consensus assembly is a mix of sequences retrieved from different homeologs (parental subgenomes) and chimeric sequences. Therefore, the crucial steps to resolve in polyploid phylogenetics are to separate sequencing reads originating from different subgenomes, assemble haplotype (allele) sequences, assign them to the subgenomes, and trace the parental origin of these subgenomes by multilabeled species tree or network inference methods (Rothfels, 2021). A few recent studies have explored different ways how to accomplish these steps, either via mapping and categorization of the sequence reads to the reference diploid genomes (Page et al., 2013; Grover et al., 2015), developing bioinformatics pipelines for amplicon sequences of polyploids from long-read sequencing platforms (Rothfels et al., 2017), or via the assembly of haplotype sequences by read-backed phasing (Eriksson et al., 2018; Kates et al., 2018). Nevertheless, the assignment of alleles to parental subgenomes has been critical and difficult to achieve readily for hundreds of loci typically recovered by target enrichment techniques. Some statistical methods for this task are under development and appear promising (Freyman et al., 2020; Lautenschlager et al., 2020), but may also be computationally intensive.

In this article, we employ target enrichment with genome skimming (Hyb-Seq) using genus-specific probes to capture hundreds of orthologous low-copy nuclear loci (target exons with flanking intronic and intergenic regions), along with obtaining the complete plastid genome and high-copy nuclear ribosomal DNA (Weitemier et al., 2014; Schmickl et al., 2016). Here we develop a novel computational approach to sort alleles obtained from polyploids into parental subgenomes, utilizing genetic distances among alleles, and employ it to reconstruct the origin and parentage of tetraploid *C. barbaraeoides*. We complement this phylogenomic approach with genomic *in situ* hybridization (GISH, Silva and Souza, 2013). In detail, we aimed to (1) resolve phylogenetic relationships among Balkan *Cardamine* species and determine major factors affecting endemism patterns in mountains of the Balkan Peninsula; (2) reconstruct the origin of tetraploid *C. barbaraeoides* from the Pindos Mts. in Greece to shed light on the evolution of mountain endemic flora through polyploidy; and (3) identify challenges of phylogenomic analyses of polyploid species, where we focus on resolving heterozygous and homeologous sequence variation and its sorting into parental subgenomes.

## Materials and Methods

### Study Species and Sampling

**FIGURE 1:**
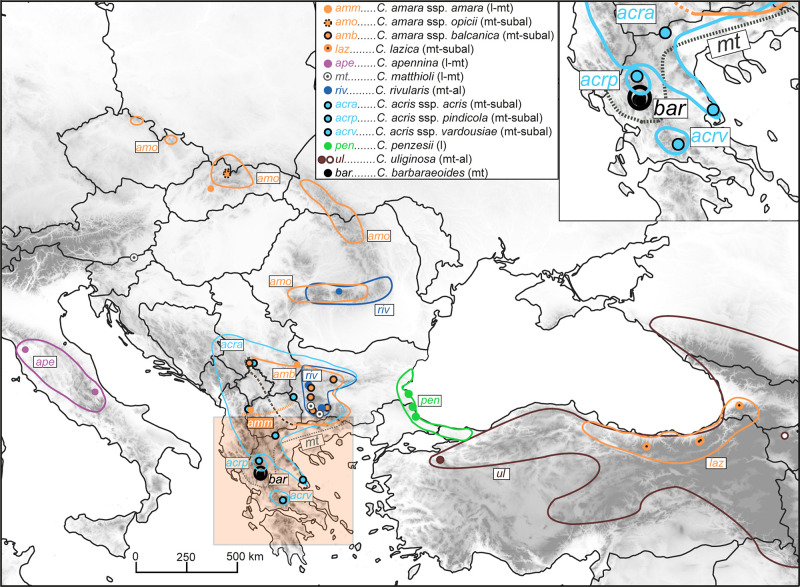
Distribution of the *Cardamine* taxa under study, based on data compiled from floras, herbarium specimens, previous studies, and our own records. The western borders of the area of *Cardamine amara* subsp. *balcanica* remain unknown (marked here by a black–orange dashed line; see Tomović et al., 2009); this taxon has been thoroughly studied so far only in its Bulgarian range (Marhold et al., 1996). *Cardamine matthioli* and *C. amara* subsp. *amara* are widespread taxa in Europe (Jalas and Suominen, 1994), but their precise distribution in the Balkan Peninsula remains unclear, with the southernmost records reported from central and northeastern Greece (gray dotted line; Marhold and Tan, 2000) and North Macedonia (orange dotted line; Jalas and Suominen, 1994; Tomović et al., 2009, and this study), respectively. The area of *Cardamine uliginosa* extends further to the south and southeast, reaching the mountains of Iran and Lebanon. The occurrence of the taxa along the elevational gradient is indicated in brackets as follows: l, lowland; mt, montane; subal, subalpine; al, alpine belt. Circles indicate our sample sites; see Supplementary Data Sheet 1 for details on the populations sampled.

The target species complexes of *Cardamine* comprise rhizomatous perennials with an allogamous or mixed mating system, capable of vegetative propagation (Lövkvist, 1956; Marhold and Ančev, 1999; Tedder et al., 2015). They grow in wet habitats from lowlands up to the alpine belt, in or nearby running or standing water, usually along river and stream banks, in springs, wet meadows and pastures, in flood-plain to montane forests. Morphologically, they are characterized by pinnate basal leaves, pinnate to pinnatisect stem leaves, and white, pale pink to purple flowers arranged in racemes (e.g., Marhold et al., 1996; Marhold and Ančev, 1999; Lihová et al., 2004b; Perný et al., 2004). In the Balkan Peninsula, they include mostly endemics (*C. amara* subsp. *balcanica*, *C. acris* subsp. *acris*, subsp. *vardousiae* Perný et Marhold, subsp. *pindicola* Perný et Marhold, *C. barbaraeoides*, *C. penzesii*, *C. rivularis*) or more widespread European taxa reaching their southeastern distribution margins there [*Cardamine matthioli* Moretti, *C. amara* subsp. *amara*, subsp. *opicii* (J. Presl et C. Presl) Čelak; Figure 1]. Apart from tetraploid records for *C. barbaraeoides* (Perný et al., 2005a; Lihová and Marhold, 2006], the other Balkan taxa are known to be diploid, with exceptional triploid plants reported for *C. rivularis* and *C.* ×*rhodopaea* Ančev (*C. rivularis* × *C. matthioli*) (Kučera et al., 2005; Ančev et al., 2013; Melichárková et al., 2020). Only diploid representatives have so far been reported from the adjacent Anatolian–Caucasus region (Marhold et al., 2004; Kučera et al., 2005). In the Apennines, on the contrary, one diploid (*Cardamine apennina* Lihová et Marhold) and two polyploids (*Cardamine silana* Marhold et Perný, *Cardamine amporitana* Sennen et Pau, both presumably allopolyploids, Perný et al., 2005a, and unpubl. results) occur (Marhold et al., 2018). The three species, *C. amara*, *C. amporitana*, and *Cardamine lazica* Boiss. et Balansa ex Buser (the last one being referred to as *Cardamine wiedemanniana* Boiss. in our previous studies; see, e.g., Lihová et al., 2004a), have been regarded as members of the *C. amara* complex, whereas the other species have been attributed to either the *C. pratensis* or *C. raphanifolia* groups. The position of *C. barbaraeoides* remained uncertain and was commonly classified either as *C. amara* subsp. *barbaraeoides* (Halácsy) Maire et Petitm. (Tan, 2002) or as *C. raphanifolia* subsp. *barbaraeoides* (Halácsy) Strid (Strid, 1986; Jones and Akeroyd, 1993).

Here, we included all taxa occurring in the Balkan Peninsula, plus diploids from adjacent areas, *C. apennina* from the Apennines, and *C. lazica* and *Cardamine uliginosa* M.Bieb. from the Anatolian–Caucasus region (Figure 1, Supplementary Data Sheet 1). *C. uliginosa* is a highly polymorphic and widespread species (Figure 1) described from the Caucasus, but probably being polyphyletic and pending further detailed studies (Marhold et al., 2004; study under progress). Two geographically distant accessions attributed to this species were included here, one from the Uludağ Mts. in NW Turkey, here referred to as *C.* cf. *uliginosa*, and the other from the Caucasus (Armenia). Altogether, we sampled 46 populations representing nine species (13 taxa), which were used for ploidy level and genome size measurements by flow cytometry (307 accessions), polymerase chain reaction (PCR) amplification of the nuclear ribosomal DNA (nrDNA) ITS region (48 accessions), and Hyb-Seq analyses (22 accessions) capturing target nuclear genes, plastid DNA, and nrDNA. The tetraploid *C. barbaraeoides* and a selection of potential parental candidates were used in GISH experiments. In addition, four diploids representing phylogenetically divergent lineages (following the genus phylogeny, Carlsen et al., 2009) were included as outgroups. The list of the populations sampled and accessions analyzed is given in Supplementary Data Sheet 1.

### Chromosome Counting and Flow Cytometry

Chromosomes of *C. barbaraeoides* were counted from mitotic metaphase plates observed in cells of young, actively growing root tips obtained from cultivated plants. Chromosome spreads were prepared following Marhold et al. (2002) using the Giemsa stain, or following Mandáková and Lysak (2016a) using the DAPI (4′,6-diamidino-2-phenylindole) fluorochrome. For the other sampled species and subspecies, chromosome number records were available from previous studies (Kučera et al., 2005), in some cases even from the here sampled localities (see Supplementary Data Sheet 1 for details).

Flow cytometry was applied here to measure nuclear DNA content of the sampled accessions (Doležel et al., 2007). These measurements were performed to confirm that the ploidy level of the analyzed populations and accessions is uniform and agrees with the known records, as well as to determine genome size differences between the species. Both absolute and relative nuclear DNA content was measured, using the DNA-intercalating fluorochrome [propidium iodide (PI)], and the AT-selective DAPI fluorochrome, respectively (Doležel et al., 2007). For PI measurements, we used fresh leaf tissue from cultivated plants, whereas for DAPI measurements we used silica gel–dried tissue (Suda and Trávníček, 2006a, b). Each individual was analyzed separately (for precise relative or absolute nuclear DNA content values), or up to three individuals were pooled (for ploidy level inference only; see Supplementary Data Sheet 1). Sample preparation followed the protocols described by Marhold et al. (2010). Fluorescence of the stained nuclei was measured using Partec CyFlow flow cytometers (Partec GmbH, Münster, Germany), either with a UV LED lamp for DAPI measurements or a green solid-state laser for PI measurements. Relative nuclear DNA content (2C value given in arbitrary units) was calculated as the ratio between the positions of the G1 peaks of the sample and the standard. Absolute nuclear DNA content (2C value given in pg) was calculated from the ratio of the respective G1 peaks and the known 2C value of the standard. *Solanum pseudocapsicum* (2*C* = 2.59 pg; Temsch et al., 2010) was used as the primary internal standard. In cases when peak overlaps between the sample and standard were observed or expected, *Bellis perennis* (2*C* = 3.38 pg; Schönswetter et al., 2007) was used as the secondary standard (see Supplementary Data Sheet 1).

### PCR Amplification, Molecular Cloning, and Sanger Sequencing

Polymerase chain reaction amplification, molecular cloning, and Sanger sequencing of the ITS region of nrDNA were employed to explore the diversity of ITS variants within and between individuals, both diploid and tetraploid, as well as to compare this approach with the accuracy and efficiency of retrieving different ITS variants from high-throughput genomic reads. In addition, PCR amplification, molecular cloning, and Sanger sequencing of *chalcone-synthase* (*CHS*) was performed for tetraploid accessions only (Supplementary Data Sheet 1). *CHS* is a single-copy nuclear gene of high phylogenetic resolution, used previously to infer polyploid origins and phylogeny of *Cardamine* species (Lihová et al., 2006; Kučera et al., 2010). It was included among the target genes in the Hyb-Seq approach, and therefore, the sequenced *CHS* clones were used to verify and optimize the assembly of allele sequences by read-backed phasing and the procedure of allele sorting into parental homeologs in tetraploid accessions (see below).

Genomic DNA (gDNA) was isolated from silica gel–dried leaves using the DNeasy Plant Mini Kit (Qiagen, Germany) or GeneAll Exgene Plant SV mini kit (GeneAll Biotechnology Co., LTD., South Korea). ITS amplifications and molecular cloning followed the protocols specified in Melichárková et al. (2017, 2019). Exon 2 of *CHS* was amplified with the primers CHSF2 and CHSR1 (Lihová et al., 2006) and cloned following Melichárková et al. (2017). The PCR reaction mix contained also 3% dimethyl sulfoxide to suppress PCR-mediated recombination events. Multiple clones per sample were sequenced (see Supplementary Data Sheet 1 for details). The sequencing was carried out at Eurofins Genomics Company (Konstanz, Germany).

### Hyb-Seq Library Preparation

Sequencing libraries were prepared using the NEBNext^®^ Ultra^TM^ DNA Library Prep Kit for Illumina^®^ (New England Biolabs, MA, United States) following the manufacturer’s protocol. gDNA (400 ng per accession) was fragmented with a Covaris M220 sonicator (Woburn, MA, United States) to a target fragment size of 500 bp. Adaptor-ligated DNA fragments were purified with the QIAquick PCR Purification Kit (Qiagen) and size-selected using SPRIselect beads (Beckman Coulter, MA, United States) to a 500- to 600-bp size range. PCR enrichment with eight cycles was performed using index primers from NEBNext^®^ Multiplex Oligos for Illumina^®^. The amplified libraries were cleaned up with AMPure XP beads (Beckman Coulter), measured with a Qubit 2.0 fluorometer (ThermoFisher Scientific, MA, United States), and pooled equimolarly (24 accessions/pool). The pooled library was size-selected using SPRIselect beads as above and measured again with the Qubit 2.0. An aliquot containing 250 ng was enriched by hybridization with synthesized RNA baits (26 h at 65°C) using the MYbaits^®^ kit, following the protocol v. 3.02 (Arbor Biosciences, MI, United States). The target-enriched library was amplified by PCR with nine cycles using the KAPA HiFi HotStart mix (Kapa Biosystems, Wilmington, MA, United States) and purified with the QIAquick PCR Purification Kit. Enriched and unenriched library aliquots were pooled in a ratio 2:1, finally purified with AMPure XP beads, and submitted for sequencing with 150-bp paired end reads on an Illumina MiSeq system at BIOCEV, Czechia.

The design of the *Cardamine*-specific target enrichment probes is described in detail in Melichárková et al. (2020). In brief, we used genome skim data of *Cardamine parviflora* (NCBI accession no.: SRR11977919) omitting plastid and mitochondrial reads, which were matched against unique transcripts of *C. amara* (SRR11977918), utilizing the workflow of the Sondovač 0.99 script^1^ (Schmickl et al., 2016). Genome skim hits were assembled into larger contigs, which were filtered for length and uniqueness, and compiled as probe sequences for bait synthesis. In total, 14,464 120-mer biotinylated RNA baits, capturing 2,246 exons from 1,235 genes, were synthesized by MYcroarray (now Arbor Biosciences).

### Hyb-Seq Data Processing and Phylogenomic Analyses

Demultiplexed reads were trimmed of adapters and low-quality bases using Trimmomatic v. 0.36 (Bolger et al., 2014). Read ends with quality below Q20 were discarded, and the remaining part of the read was trimmed if average quality in a 4-bp sliding window was below Q15. Finally, any reads trimmed to less than 50 bp were discarded. PCR duplicates were removed using the Clumpify command of BBTools^2^.

Consensus target sequences were assembled using HybPiper version 1.3 (Johnson et al., 2016) utilizing BWA v. 0.7.13 (Li and Durbin, 2009), SPAdes v. 3.13 (Bankevich et al., 2012), and Exonerate v. 2.2 (Slater and Birney, 2005). HybPiper generates a single consensus sequence per individual, with potentially heterozygous bases called as the nucleotide with the highest read frequency. “Supercontigs” (targeted exons and flanking sequences) were recovered using the script intronerate.py. Recovered consensus supercontig sequences were aligned using MAFFT v. 7.313 (Katoh and Standley, 2013). Flanks and sites with gaps in more than 25% of sequences were removed using the ips R package (Heibl, 2008 onward) in R 3.3.2 (R Core Team, 2019). Alignments were inspected visually, and misassemblies were removed. In addition to using the consensus supercontig sequences, the allele sequences were inferred with read-backed phasing (described in detail below in *Extracting Allele Sequences and Identifying Homeologs Inherited From Different Parents*) using WhatsHap (Martin et al., 2016). Both consensus and allele data sets were used in further analyses.

The recovered sequences of the target nuclear genes were analyzed using the following workflow. First, we performed phylogenomic analyses of diploid taxa only (with both the consensus and allele sequence alignments), to provide a robust phylogenetic framework, using both concatenation of assembled genes and species tree inference under the multispecies coalescent model. As next, we analyzed diploids together with the tetraploid *C. barbaraeoides*. Considering that the tetraploid genome consists of two subgenomes that may be more or less differentiated, and thus potentially conveys conflicting phylogenetic information, we used here multiple approaches. To gain initial insights into the tetraploid genome, we used consensus supercontig sequences and applied methods that can detect and visualize conflict caused by potential discordance between consensus supercontigs retrieved from independent genes. In allopolyploids, the consensus sequences may comprise different homeologs or even consist of artificial, chimeric sequences. The analyses included supernetwork and species network calculations based on the gene trees obtained from the assembled consensus sequences, as well as single-nucleotide polymorphisms (SNPs) calling followed by Bayesian clustering of the SNP datasets. Finally, when the conflict between the subgenomes of the tetraploid became apparent, we derived allele sequences of the exons by read-backed phasing also from the tetraploids (see below in *Extracting Allele Sequences and Identifying Homeologs Inherited From Different Parents*). Up to four different alleles obtained from the exons of tetraploid *C. barbaraeoides* were sorted into two distinct homeologs based on allelic divergence (computing interallelic distances, see below) using an optimized threshold value. The resulting allele alignments were submitted to coalescent-based species tree inference.

Phylogenetic trees were constructed using RAxML-NG v. 0.9.0 (Kozlov et al., 2019). The best-fit model of substitution for each gene, exon, or partitioning scheme was estimated using the IQ-TREE’s ModelFinder function (Chernomor et al., 2016; Kalyaanamoorthy et al., 2017) under the Bayesian information criterion. Branch support of the best ML trees was estimated by 500 bootstrap (BS) replicates. The quartet sampling method (Pease et al., 2018), which can distinguish strong conflict from weak signal, was applied to assess branch support of the trees generated from the concatenated alignments. The concatenation of the aligned exons and genes was performed by AMAS (Borowiec, 2016). Species trees were inferred from individual gene trees under a multispecies coalescent model using ASTRAL-III (Zhang et al., 2018). PhyloNet was employed to infer a species network evaluating reticulate evolutionary relationships in individual gene trees. The network was inferred with a single reticulation node using the InferNetwork_MP method in 10 runs, each with two optimal networks returned (Wen et al., 2018). SuperQ v.1.1 (Grünewald et al., 2013; Bastkowski et al., 2018) decomposed gene trees into quartets, and inferred a supernetwork selecting the JOptimizer scaling and Gurobi optimizer. The trees used as input data for species tree reconstruction and both network analyses had contracted branches with low support (≤ 20%) by Newick-Utilities v. 1.6 (Junier and Zdobnov, 2010). Bayesian clustering of SNP data was performed to infer homogeneous genetic clusters with STRUCTURE 2.3.4 (Pritchard et al., 2000). Input datasets were generated by the snipStrup pipeline [available online at: https://github.com/MarekSlenker/snipStrup; described in detail in Melichárková et al. (2020)]. This pipeline uses target sequences (those used for probe synthesis) as a reference and calls variants with respect to ploidy. To ensure that no linkage existed between sites, 500 datasets were produced by drawing a single random SNP site from each gene containing at least 10 SNPs across the samples. Each dataset was run for each *K* = 1–10 (user-defined number of clusters), with a burn-in length of 100,000 generations and data collection for an additional 900,000 generations, setting the admixture model and correlated allele frequencies. The results of 500 datasets were averaged using the program CLUMPP (Jakobsson and Rosenberg, 2007) and drawn with Distruct (Rosenberg, 2004). The approach of Evanno et al. (2005) was used to determine the optimal *K* value.

### Extracting Allele Sequences and Identifying Homeologs Inherited From Different Parents

Allele sequences were derived using the scripts and following the workflow available online at: https://github.com/mossmatters/phyloscripts/tree/master/alleles_workflow, described in detail by Kates et al. (2018), only using the latest versions of GATK and WhatsHap (Martin et al., 2016; Schrinner et al., 2020) enabling to call and phase variants in polyploids. If the phased sequences were divided into multiple blocks, only the longest phase block for each individual was retained, and the remaining interallelic variant sites were masked by using Ns on those positions.

The alleles obtained from the tetraploid *C. barbaraeoides* were sorted into two distinct homeologs as follows. The first step was to find two pairs of alleles, in which the alleles are closest to each other within the pairs while more distant between the pairs. Interallelic distances were estimated from the branch lengths of the corresponding exon or gene ML trees (computed by cophenetic function of package stats, R Core Team, 2019). The optimal threshold for unequivocal allele sorting was set to 4 (for more details about searching for the optimal threshold value, see Supplementary Text 1). This means that if an average distance between alleles within the proposed two pairs was more than four-time shorter than the average distance between alleles within any other possible arrangement, these pairs of alleles were considered unequivocally different and attributable to different homeologs (see also Supplementary Text 2). If the allele sorting did not pass the desired threshold, two options were followed. Either the interallelic SNPs were masked by using Ns on those positions (such unsorted, masked exons were used for further concatenation into gene alignments, see below) or the sample was removed from the alignment (for exon-based analyses). As next, the allele pairs were attributed to different homeologs and labeled by calculating their distances to the alleles of all diploid species. The allele pair that was closer to *C. amara* (proposed as the maternal parent according to the plastome phylogeny, see below) was marked as homeolog “A”, and the other pair as homeolog “B”. Gene alignments were also assembled, in which the phased alleles of the respective exons were concatenated to genes to obtain longer alignments with potentially stronger phylogenetic signal. The concatenated exons included those with successfully sorted alleles into “A” and “B” homeologs and those for which allele sorting was equivocal, with masked interallelic SNPs. After exon concatenation, the allele sorting into two homeologs was verified for each gene, with the same threshold as set for the exons above, to confirm unambiguity or to remove the equivocal sample from the gene alignments. Both exon-based and gene-based alignments were used for species tree inference in ASTRAL-III. The labeled homeologs, representing the two subgenomes within *C. barbaraeoides*, were treated as independent accessions. The scripts used are available online at: http://github.com/MarekSlenker/AlleleSorting.

### Gene Genealogy Interrogation Analyses

To explore the significance of phylogenetic placements of the A and B homeologs of *C. barbaraeoides*, we performed alternative topology testing using the gene genealogy interrogation (GGI) analyses (Arcila et al., 2017). This approach accounts for gene tree estimation error and evaluates the relative support for specific alternative hypotheses. First, the hypotheses to be tested are defined by performing constrained ML gene tree searches with enforced monophyly of the examined clades in RAxML. Here we considered three different topologies for both A and B homeologs, following the results of PhyloNet analyses and exon- and gene-based species trees inferred from phased sequences (see *Results* for details). The topology test was then performed for each nuclear gene or exon (i.e., considering both exon- and gene-based phased datasets) by statistically comparing the site likelihood scores obtained for each constrained tree in RAxML using the approximately unbiased (AU) topology test implemented in CONSEL (Shimodaira and Hasegawa, 2001; Shimodaira, 2002). The AU test performs simultaneous comparisons of multiple trees and estimates a *P* value for each topology. The trees are then ranked according to the *P* values, and the results are visualized as plots of the cumulative number of constrained gene trees and their AU test *P* values for each topology.

### Analyses of nrDNA Sequence Data

nrDNA sequences obtained from molecular cloning were aligned in Geneious v. R10 (Kearse et al., 2012). Sequences of nrDNA were also recovered from Hyb-Seq data in HybPiper using *C. amara* (AY260579.1) and *C. pratensis* (KF987809.1) reference sequences, as specified above for the target nuclear loci, but omitting the “supercontig” option. The sequences were aligned using MAFFT v. 7.450 (Katoh and Standley, 2013), and only the ITS region was extracted and kept for further analyses to allow for direct comparison with the cloned data. The sequences recovered from HybPiper were also proceeded further to read-backed phasing to retrieve multiple nrDNA variants, as described above. Here were generated four nrDNA datasets as follows: (1) alignment obtained from molecular cloning; (2) consensus assembly with base calling following the majority rule criterion, as produced by HybPiper; (3) ambiguous assembly with intraindividual SNPs replaced by IUPAC codes produced by bcftools consensus command; and (4) “multiallelic” (read-backed phasing) alignment, where multiple nrDNA variants were retrieved for each sample. Maximum likelihood (ML) trees were inferred with RAxML-NG as above.

### Analyses of Chloroplast Genome Data

Chloroplast DNA sequences were assembled using Fast-Plast v. 1.2.8 (available online at: https://github.com/mrmckain/Fast-Plast) with default settings. This pipeline utilizes Trimmomatic v. 0.39 (Bolger et al., 2014) for initial read cleaning, Bowtie 2 v. 2.3.5.1 (Langmead and Salzberg, 2012) to extract chloroplast reads using a database of reference plastomes, SPAdes v. 3.13 (Bankevich et al., 2012), and afin (available online at: https://github.com/mrmckain/Fast-Plast/tree/master/afin) for *de novo* sequence assembly. For two accessions, for which the plastome assembly failed in Fast-Plast, chloroplast DNA sequences were assembled in HybPiper using the *C. amara* (KY562580.1) reference sequence. The obtained plastome sequences, comprising the large single copy (LSC), the small single copy (SSC), and one copy of the inverted repeats (IRb), were aligned using MAFFT v. 7.450 (Katoh and Standley, 2013). Gene annotation (protein coding, tRNA and rRNA genes) was performed with GeSeq (Tillich et al., 2017). Two chloroplast DNA (cpDNA) alignments were generated and used for phylogenetic tree reconstructions, one comprising the complete sequences of the LSC, SSC, and IRb regions, including intergenic spacers, and the other consisting of the concatenated sequences of annotated genes only. ML trees were inferred in RAxML-NG as above. Although it has been widely assumed that plastid genes are inherited as a single locus, favoring their concatenation before phylogenetic analyses, some recent studies have indicated that these genes may not be as tightly linked as expected and may experience different evolutionary histories. Therefore, the application of multispecies coalescent methods to account for potential discordance between gene trees has been advocated also for plastome genes (Gonçalves et al., 2019; Walker et al., 2019). Following this research, we extracted the most variable protein-coding genes (42 genes, those > 350 bp long with > 10 variable positions in the alignment), for which separate ML gene trees were constructed in RAxML-NG. The obtained ML gene trees were then used for species tree inference in ASTRAL-III.

### Genomic *in situ* Hybridization

Genomic *in situ* hybridization was performed in *C. barbaraeoides* to identify its parental chromosome complements. GISH probes were prepared from total gDNA of eight diploid taxa, *C. acris* subsp. *acris*, *C. amara* subsp. *amara*, subsp. *balcanica*, *C. lazica*, *C. matthioli*, *C. penzesii*, *C. rivularis*, and *C. uliginosa* (see Supplementary Data Sheet 1), which were used in different combinations. Mitotic chromosome spreads of *C. barbaraeoides* were prepared as described above, following Mandáková and Lysak (2016a). To remove RNA and cytoplasm, the preparations were treated with 100 μg/mL RNase (AppliChem) in 2 × sodium saline citrate (20 × sodium saline citrate: 3 M sodium chloride, 300 mM trisodium citrate, pH 7.0) for 60 min, and 0.1 mg/mL pepsin (Sigma) in 0.01 M HCl at 37°C for 5 min, and then postfixed in 4% formaldehyde in 2 × sodium saline citrate for 10 min, washed in 2 × sodium saline citrate twice for 5 min, dehydrated in an ethanol series (70%, 80%, and 96%, 2 min each), and air-dried. gDNA of the diploids was extracted from silica gel–dried leaves using the DNeasy Plant Mini Kit (Qiagen). Isolated gDNA was labeled with either biotin-dUTP or digoxigenin-dUTP via nick translation according to Mandáková and Lysak (2016b). Individual labeled probes were stored at −20°C until use. The GISH protocol followed Mandáková et al. (2013, 2014). The immunodetection of hapten-labeled probes was performed as follows: biotin-dUTP was detected by avidin–Texas red (Vector Laboratories) and amplified by goat anti-avidin–biotin (Vector Laboratories) and avidin–Texas red; digoxigenin-dUTP was detected by mouse antidigoxigenin (Jackson ImmunoResearch) and goat anti-mouse–Alexa Fluor 488 (Invitrogen). After immunodetection, chromosomes were counterstained with DAPI (2 μg/mL) in Vectashield (Vector Laboratories). Painted chromosome figures were photographed using an Axioimager Z2 epifluorescence microscope (Zeiss) equipped with CoolCube CCD camera (MetaSystems). Images were acquired separately for the three fluorochromes using appropriate excitation and emission filters (AHF Analysentechnik). The three monochromatic images were pseudocolored, merged, and cropped using Photoshop CS (Adobe Systems) and Image J (National Institutes of Health) software.

## Results

### Chromosome Numbers and Genome Size Variation

**FIGURE 2:**
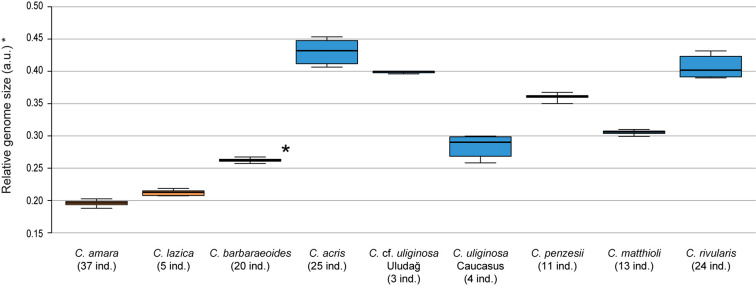
Genome size variation of the *Cardamine* species under study, based on flow cytometric analyses. Relative nuclear DNA content inferred from DAPI measurements is presented, given as the ratio of the sample and standard G1 peaks (2C values in arbitrary units, a.u.). In the tetraploid *Cardamine barbaraeoides* (marked by asterisk), however, DNA content of the meiotically reduced genome (corresponding to the 2*x* level) is assessed and presented. Population C018 of *Cardamine acris* was omitted because of its divergent DNA content and unclear ploidy level (see Supplementary Data Sheet 1). Boxplot graphs show the 25th and 75th percentiles (boxes), median values (vertical lines within boxes), and minimum to maximum values (whiskers). Orange color is used for species of the *Cardamine amara* group, blue for the other diploids, and black for the tetraploid *C. barbaraeoides*. The number of analyzed individuals per species is indicated. See Supplementary Data Sheet 1 for more details and population-level values.

Chromosome counting revealed the tetraploid level with 2*n* = 32 chromosomes in *C. barbaraeoides*, determined in two populations. Flow cytometry confirmed the tetraploid level in all five sampled populations (27 individuals in total; Supplementary Data Sheet 1). Ploidy level screening within the other studied species showed consistent results, supporting a single, diploid level. Only few exceptions were identified, such as one apparently triploid individual of *C. acris* and population C018 of *C. acris* with increased genome size values not attributable to any ploidy level with certainty (Supplementary Data Sheet 1). The diploid species displayed a wide range of 2C values, and most of the species differed from each other in their nuclear DNA content (Supplementary Data Sheet 1, Figure 2). Populations of *C.* cf. *uliginosa* from the Uludağ Mts. (UD, northwestern Turkey) and the Caucasus Mts. (AM, Armenia) showed markedly different values (in accordance with their genetic divergence, see below) and were kept as two separate entities. The smallest genome sizes were observed in *C. amara* and *C. lazica*, whereas the largest ones in *C. acris*, *C. rivularis*, and *C.* cf. *uliginosa* from the Uludağ Mts., being more than twice as big as in *C. amara*. In accordance with the tetraploid level, the largest nuclear DNA content was measured in *C. barbaraeoides*, but when recalculated to the meiotically reduced genome (corresponding to the 2*x* level), it showed an intermediate value placed among the diploids (Figure 2).

### Hyb-Seq Data

The sequencing process yielded, on average, 1.28 million reads per sample. Adapter trimming, quality filtering and deduplication resulted in an average loss of 1.06% of reads. Of the remaining reads, 54.11% on average were mapped to the target nuclear gene sequences, which ensured mean coverage of more than 97 reads per base. Mean coverage of the plastid genome fluctuated widely among samples, from 13.5 to 96.23 reads per base (43.56 on average). The same was true for the ITS region of nrDNA, but the mean coverage of all samples was more than 70 reads per base. Of the 2,246 exons from 1,235 genes, targeted by the designed RNA baits, 1,858 (82.72%) consensus sequences were assembled in all 22 samples. More than 98% of sequences, that is, 1,829 supercontigs representing 1,168 genes, passed inspection and were used for further analyses. The length of the exon alignments ranged from 63 to 3,548 bp (709 bp on average), whereas the gene length ranged from 72 to 8,458 bp, with a mean of 1,111 bases. The concatenated alignment of all genes was 1,297,401 bp long.

### Phylogenomic Analyses of Diploids Based on Target Nuclear Loci

**FIGURE 3:**
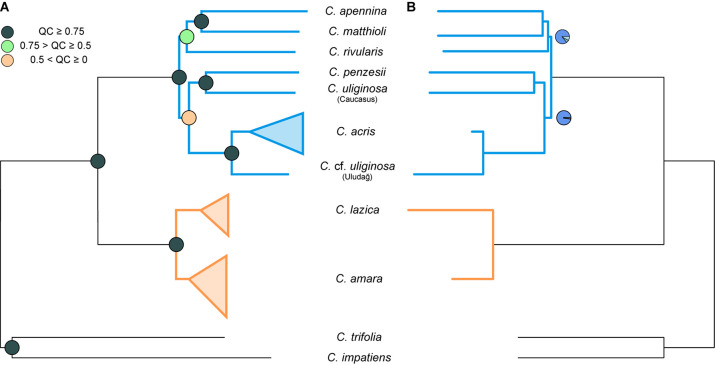
Phylogenetic trees inferred from the complete dataset of 1,168 nuclear genes, based on consensus supercontig sequences of diploid *Cardamine* accessions. Orange color is used for the clades and branches of the *Cardamine amara* group, whereas blue is used for the remaining diploids resolved in the sister position. **(A)** Maximum likelihood tree inferred in RAxML-NG from the concatenated genes. Multiple individuals per species are shown collapsed (see Supplementary Figure 1 for the fully labeled version of the tree including bootstrap support). Branch support is indicated by quartet concordance (QC) values (colored circles in the nodes). **(B)** Species tree inferred in ASTRAL-III. Branch support is indicated by pie charts, depicting three local posterior probabilities for the given branch (dark blue for the main topology as resolved here and light blue for the alternative ones; not shown for the fully supported branches when the local posterior probability for the present topology equals 1).

Maximum likelihood analysis of the diploid taxa, based on the concatenated dataset of all 1,829 loci (consensus supercontigs) from 1,168 nuclear genes, resulted in a tree with two major well-supported clades (Figure 3A, Supplementary Figure 1). One clade comprised accessions of *C. amara* and *C. lazica* in a sister position, supported by high BS as well as quartet concordance (QC) values. The other major clade exhibited a topology with strong to moderate support (QC = 0.42–1, BS = 69%–100%) and comprised three subclades as follows: (1) *C. acris* resolved in a sister position to *C.* cf. *uliginosa* from the Uludağ Mts.; (2) *C. penzesii* together with the accession of *C. uliginosa* from the Caucasus; (3) *C. apennina* and *C. matthioli* in a sister position, together with *C. rivularis*. Because the two geographically distant accessions of *C. uliginosa* (Caucasus vs. Uludağ) appeared clearly differentiated in all datasets (including nrDNA and cpDNA data, see below), they were treated as two distinct entities in all multispecies coalescent methods. The species trees inferred using ASTRAL from 1,168 ML gene trees, based either on consensus sequences (Figure 3B) or phased allele sequences (results not shown), showed identical topologies and branch support. These trees were also fully congruent with the ML tree of the concatenated dataset. Two branches that received lower QC values in the ML tree, congruently, showed slightly decreased local posterior probabilities in the species trees.

### The Tetraploid Genome of *C. barbaraeoides*: Insights From Target Nuclear Loci

#### Displaying Conflict: Network Analyses Based on Consensus Sequences and Bayesian Clustering of SNP Variation

**FIGURE 4:**
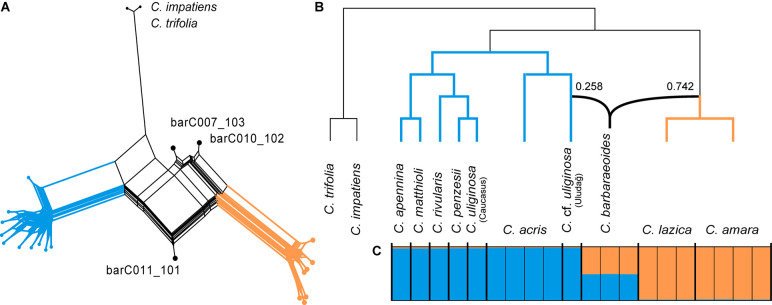
Phylogenetic analyses indicating the hybrid (allopolyploid) origin of the tetraploid *Cardamine barbaraeoides*. Orange color is used for the accessions from the *Cardamine amara* group (*C. amara* and *Cardamine lazica*), blue for the remaining diploids, and black for the tetraploid *C. barbaraeoides*. **(A)** Supernetwork representation of quartets generated in SuperQ, which was derived from 1,168 nuclear gene trees estimated in RAxML and based on consensus supercontig sequences. **(B)** Species network inferred in PhyloNet from the same set of 1,168 nuclear gene trees. Inheritance probabilities are shown along the branches indicating the origin of *C. barbaraeoides*. **(C)** Bayesian clustering of SNP datasets in STRUCTURE at optimal *K* = 2, obtained from variant calling in a selection of 947 most informative genes. The coloring in the graph indicates the sample assignment to the two genetic clusters. Thick vertical lines separate different species.

The SuperQ network derived from 1,168 ML gene trees based on consensus sequences displayed two well-differentiated groups of diploid taxa (corresponding to the two major clades as resolved above) and strong conflict in the placement of the tetraploid accessions (Figure 4A). The species network analysis (PhyloNet) based on the same set of ML gene trees suggested a hybrid origin of *C. barbaraeoides* as well, with one ancestral lineage from the clade of the *C. amara* group (comprising *C. amara* and *C. lazica*) indicating a greater inheritance probability (74.2%) and the other pointing to the *C.* cf. *uliginosa* accession from the Uludağ Mts. (25.8%), which was sister to *C. acris* (Figure 4B). Interestingly, some of the repeated PhyloNet runs indicated a reticulation event also for *C. penzesii*, involving *C.* cf. *uliginosa* from the Uludağ and the Caucasus as the two ancestors (Supplementary Figure 2).

Single-nucleotide polymorphisms calling utilized 947 genes, which harbored at least 10 SNPs across the samples. STRUCTURE analyses of 500 SNP datasets (each with one SNP randomly drawn per gene) identified the optimal genetic partitioning at *K* = 2, with the same two clusters of diploid taxa as identified in the trees above, whereas significant genetic admixture was observed in the tetraploid *C. barbaraeoides* (Figure 4C). Thus, all these analyses showed strong conflict in the consensus supercontig sequences of the tetraploid and suggested an allopolyploid origin of *C. barbaraeoides*, its progenitors being derived from the two major clades of diploids.

#### Identification of Parental Progenitors: Gene Tree and Species Tree Reconstructions Based on Phased Allele Sequences

Read-backed phasing yielded two alleles per exon for diploids and four alleles for tetraploids. In diploids, the level of heterozygosity varied widely from 10.28% to 51.34% (34.01% on average). Allele phasing in the tetraploid *C. barbaraeoides* yielded similar results among the samples. Homozygous (10.02% on average), fully heterozygous (13.5%), and partially heterozygous exons with two different alleles in the ratio 1:3 (8.4%) were relatively rare, while partially heterozygous loci with two different alleles in the ratio 2:2 (21.54%) and especially those with three different alleles (46.53%) were much more frequent (Supplementary Figure 3 and Supplementary Table 1). The complete set of 1,829 targeted exons of *C. barbaraeoides*, each phased to four alleles, was further processed to allele sorting.

The optimized threshold for allele sorting invalidated 47.64% sequences of *C. barbaraeoides*, which could not be sorted unequivocally. They definitely regarded the homozygous exons and partially heterozygous one (those with the alleles in the ratio 1:3) and part of the other heterozygous exons (Supplementary Table 1). Alleles from all three samples of *C. barbaraeoides* were successfully attributed to the A and B homeologs only in 612 exons (33.46%), but on the other hand, more than 70% of exons (1,287) kept at least one sample with successfully sorted alleles and thus held at least partial information available for coalescent-based tree reconstruction. At the gene level (with concatenated exons), attempts to sort the alleles into two different homeologs succeeded in 38.13% of sequences. Alleles from all three samples of *C. barbaraeoides* were successfully attributed to A and B homeologs in 274 genes (23.46%), and those from at least one sample were present in 621 genes (53.17%).

**FIGURE 5:**
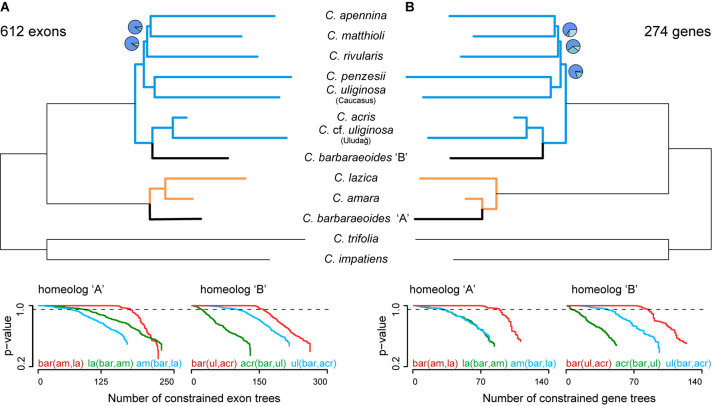
Phylogenetic analyses based on phased allele sequences of 612 exons **(A)** or 274 genes **(B)**, for which alleles of all three accessions of *Cardamine barbaraeoides* were successfully phased and sorted into A and B homeologs. Species trees were inferred in ASTRAL-III. Branch support is indicated by pie charts, depicting three local posterior probabilities for the given branch (not shown for the fully supported branches). Tree branches are colored according to the group membership; branches in black show *C. barbaraeoides*. The plots below the trees show GGI analyses, testing different phylogenetic placements of the A and B homeologs of *C. barbaraeoides*. The plots depict the cumulative number of constrained gene trees, which support the given topology, and their *P* values obtained from the approximately unbiased (AU) tests. Curves above the dashed lines indicate the number of trees that support the given topology significantly better (*P* ≤ 0.05) than the alternative ones. The tested topologies were as follows: homeolog A, red: *C. barbaraeoides* “A” being sister to the clade of *Cardamine amara* and *Cardamine lazica*, green: *C. lazica* being sister to the clade of *C. amara* and *C. barbaraeoides* “A”, blue: *C. amara* being sister to the clade of *C. barbaraeoides* “A” and *C. lazica*; homeolog B, red: *C. barbaraeoides* “B” being sister to the clade of *Cardamine acris* and *Cardamine* cf. *uliginosa* from the Uludağ Mts., green: *C. acris* being sister to the clade of *C. barbaraeoides* “B” and *C.* cf. *uliginosa* from the Uludağ Mts., blue: *C.* cf. *uliginosa* from the Uludağ Mts. being sister to the clade of *C. barbaraeoides* “B” and *C. acris*. Species trees and GGI plots from the alternative datasets allowing for missing tetraploid accessions are presented in Supplementary Figure 4.

Subsequently, for species tree inferences in ASTRAL, we assembled multiple datasets that were derived from phased exon- and gene-based alignments. For exons, they included the following: No. 1, a dataset comprising all 1,829 exons with zero to three tetraploid accessions retained for each exon (i.e., a dataset with missing accessions allowed); No. 2, a dataset comprising 974 exons each with at least two tetraploid accessions (a dataset allowing at most one accession missing); and No. 3, a dataset comprising 612 exons, in which all three tetraploid accessions were retained for each exon. The species trees inferred from all three datasets recovered the same topology and differed only in some branch support values (Figure 5A, Supplementary Figures 4A–C). As for the diploid taxa, the topology was largely congruent with that of the trees derived from the diploid sequence data only (Figure 3, see above), differing only in the placement of the species pair *C. penzesii–C. uliginosa* from the Caucasus. The position of this species pair, however, received a relatively low QC value in the tree of diploids (Figure 3A). The A homeolog of *C. barbaraeoides* was resolved in a sister position to the *C. amara* clade, comprising *C. amara* and *C. lazica*. The B homeolog of *C. barbaraeoides* was placed in a sister position to the clade consisting of *C. acris* and *C.* cf. *uliginosa* from the Uludağ (Figure 5A).

Similarly, as for the exons, three datasets of phased gene-based alignments were assembled: No. 1, a dataset comprising all 1,168 genes with zero to three tetraploid accessions retained for each gene; No. 2, a dataset comprising 441 genes each with at least two tetraploid accessions; and No. 3, a dataset comprising 274 genes, in which all three tetraploid accessions were retained for each gene. The species trees recovered the same topology for all three datasets, with differences only in branch support (Figure 5B, Supplementary Figures 4D–F), and were almost identical to those inferred from exon-based data. The only difference was in the placement of the A homeolog of *C. barbaraeoides*, which was resolved here in a sister position to *C. amara* (and not to the whole *C. amara* clade as above in exon-based trees).

When computing distances between the alleles retrieved from *C. barbaraeoides* and successfully sorted into A and B homeologs and the alleles of each diploid species, it becomes apparent that the A homeolog is closest to *C. amara* alleles, tightly followed by those of *C. lazica*, whereas the B homeolog is closest, almost equally, to the alleles of *C.* cf. *uliginosa* from the Uludağ and those of *C. acris* (Supplementary Figure 5).

#### Alternative Topology Testing: GGI Analyses

Topology tests based on the GGI analyses yielded robust and highly congruent results both from the exon- and gene-based datasets, when considering the set of trees in which alleles from all three accessions of *C. barbaraeoides* were present (i.e., successfully phased and sorted, 612 exons or 274 genes). The GGI results clearly favored the topology in which *C. barbaraeoides* homeolog A was resolved in a sister position to the clade of the *C. amara* group (Figure 5). This topology was significantly supported by a greater number of genes and exons than the alternative topologies (*P* < 0.05) and agrees also with the exon-based ASTRAL species tree. Two alternative topologies, i.e., with *C. barbaraeoides* homeolog A being sister to either *C. amara* (as seen on the gene-based species tree, Figure 5B) or *C. lazica*, received much less support. As for the placement of the B homeolog of *C. barbaraeoides*, the GGI analyses favored the topology in which *C. barbaraeoides* was placed in a sister position to the clade comprising *C. acris* and *C.* cf. *uliginosa* from the Uludağ, in accordance with the ASTRAL species trees. The second topology, with *C. barbaraeoides* being sister to *C. acris*, was significantly supported by a much smaller number of trees, followed by the third topology (*C. barbaraeoides* sister to *C.* cf. *uliginosa* from the Uludağ, suggested by PhyloNet) with only negligible support (Figure 5).

Slightly different and also equivocal GGI results in some cases were obtained when including also the exons or genes, in which one or two accessions of *C. barbaraeoides* were missing (i.e., one individual kept at minimum) because of failed allele sorting (1,287 exons or 621 gene in total). In those datasets, the two topologies with *C. barbaraeoides* homeolog B being sister either to *C. acris* or to the clade of *C. acris* and *C.* cf. *uliginosa* from the Uludağ received similar support, and none of them could be strongly favored over the other (Supplementary Figure 4). The placement of homeolog A in the dataset of 1,287 exons also remained equivocal, with similar support given for its sister position to either *C. amara* or to the clade of the *C. amara* group (comprising also *C. lazica*). In the dataset of 621 genes, the same topology for *C*. *barbaraeoides* homeolog A was favored as in the dataset of 274 genes (Supplementary Figure 4).

### Analyses of nrDNA Polymorphisms Obtained From Molecular Cloning and Genome Skim Data

**FIGURE 6:**
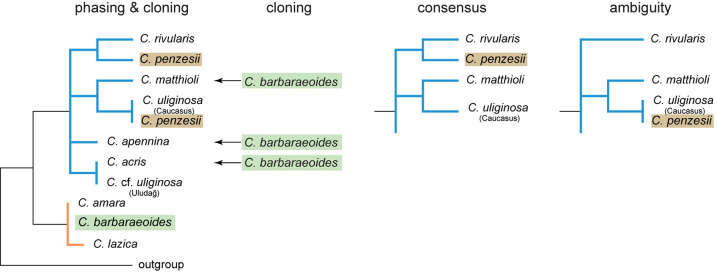
Schematic representation of maximum likelihood (ML) trees inferred from four datasets of nrDNA (IDS1-5.8S-ITS2 region) and their topological differences. Cloning: dataset obtained from molecular cloning and Sanger sequencing; phasing: dataset obtained from read-backed phasing of Hyb-Seq reads, with multiple nrDNA variants retrieved per sample; consensus: consensus assembly (with the majority rule base calls) from Hyb-Seq reads; ambiguity: ambiguous assembly from Hyb-Seq reads with intraindividual SNPs replaced by IUPAC codes. See Supplementary Figure 6 for the complete ML trees obtained from these datasets.

The ITS alignment obtained from molecular cloning was 623 bp long and comprised 180 sequences from 48 ingroup individuals. It contained 209 variable sites (33.5%) and 99 parsimony-informative sites (15.9%). High intraspecific and even intraindividual diversity of the ITS variants (ribotypes) was revealed in the diploid taxa (Supplementary Data Sheet 1). Nevertheless, the ribotypes observed within individuals and within species were mostly similar and clustered together, with the exceptions of rare divergent ribotypes found in a single accession of *C. acris* (C015-107) and *C. penzesii* (DEM7) (Supplementary Figure 6). In accordance with the data from the target nuclear loci, genetic differentiation was observed within *C. uliginosa*; ribotypes from the Uludağ samples were nested within the diversity of *C. acris*, whereas those from the Caucasus appeared closest to *C. penzesii* or *C. matthioli* (Figure 6, Supplementary Figure 6). In the tetraploid *C. barbaraeoides*, the vast majority (approximately 78%) of ITS sequences were placed within the *C. amara* clade. Three ribotypes (i.e., 4.6%) of *C. barbaraeoides* (found in three different accessions), however, were clearly divergent and clustered closest to *C. acris*, *C. matthioli*, or *C. apennina* (Figure 6, Supplementary Figure 6). The rest of the ribotypes (17.4%) showed recombinant patterns between the two major clades (not included in the ML tree).

The ITS alignment from the consensus assembly of the reads mapping to the reference sequence comprised 20 ingroup sequences with 53 variable (8.5%) and 30 parsimony-informative sites (4.8%). The alignment of the ambiguous assembly contained 68 ambiguous bases and 43 variable (6.9%) and 28 parsimony-informative sites (4.5%). Read-backed phasing of the assembled ITS sequences resulted in 1 to 4 ITS variants per individual, and the alignment comprised 47 different ingroup sequences with 77 variable (12.4%) and 57 parsimony-informative sites (9.2%). The topologies of the ML trees obtained from the consensus and ambiguous datasets were largely congruent (Figure 6, Supplementary Figure 6), except of the position of *C. penzesii*. In the consensus dataset, *C. penzesii* was resolved as sister to *C. rivularis*, whereas in the ambiguous dataset it was placed sister to *C. uliginosa* from the Caucasus. The former topology agreed with the position of all but one ribotype resolved in *C. penzesii* by molecular cloning, whereas the latter corresponded to the position of one divergent ribotype revealed in this species. In both the consensus and ambiguous datasets, the tetraploid *C. barbaraeoides* was placed within the *C. amara* clade. Phasing revealed the presence of divergent nrDNA variants in both *C. acris* (accession C015-107) and *C. penzesii* (DEM7) that were placed outside of the respective species-specific clades, being in congruence with the cloned data (Figure 6, Supplementary Figure 6). In the tetraploid *C. barbaraeoides*, by contrast, with phasing using GATK and WhatsHap tools as described above, we were able to retrieve only nrDNA variants corresponding to the *C. amara* sequence types. The rare ribotypes clustering with *C. acris*, *C. matthioli*, or *C. apennina* as found by cloning could not be successfully extracted, although visual exploration of the genomic data (using IGV; Robinson et al., 2011) confirmed the presence of a low proportion SNPs (approximately 10%) suggesting that these rare sequence variants are indeed present in the genome of *C. barbaraeoides*.

### Analyses of Chloroplast Genome Data

The alignment of the complete LSC, SSC, and IRb regions was 128,344 bp long. The alignment of the concatenated annotated genes was 96,838 bp long and included 74 protein-coding genes and 31 tRNA and four rRNA genes. The ML trees inferred from the two alignments showed high congruence (Supplementary Figure 7). Topological differences were found only in clades that displayed very short branches and low BS support. Two major clades with high BS were retrieved in both ML trees, which corresponded to those resolved by nuclear genes. One comprised *C. amara* and *C. lazica* in a sister position, which were successively sister to *C. barbaraeoides*. The other major clades in the ML trees comprised two well-differentiated and supported subclades. One subclade consisted of *C. acris* (three out of four accessions) in a sister position to *C.* cf. *uliginosa* from the Uludağ, in concordance with the topology retrieved from nuclear genes. The other subclade comprised *C. apennina*, *C. penzesii*, one accession of *C. acris* (C015), *C. rivularis*, and *C. matthioli*. Except of the last two species, resolved in a well-supported sister position, the relationships within this subclade received only low support and differed between the two cpDNA datasets.

**FIGURE 7:**
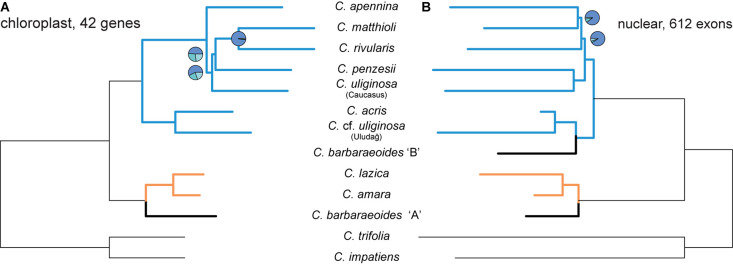
Species trees inferred in ASTRAL-III from the dataset of 42 protein-coding most informative chloroplast genes **(A)** and 612 nuclear exons [**(B)**, as in Figure 5A]. Branch support is shown by pie charts, depicting three local posterior probabilities for the given branch (not shown for the fully supported branches). Tree branches are colored according to the group membership; branches in black show *Cardamine barbaraeoides*.

The ASTRAL species tree based on 42 most variable protein-coding chloroplast genes (Figure 7A) showed high congruence with the ML trees inferred from the concatenated alignments (Supplementary Figure 7A). Two internal branches, which determined the positions of *C. penzesii* and *C. uliginosa* from the Caucasus, received low local PP values that imply low support for the given topology. This agrees with the topological differences between the ML trees from the concatenated data and thus suggests that the placement of these two species is uncertain.

### Genomic *in situ* Hybridization

**FIGURE 8:**
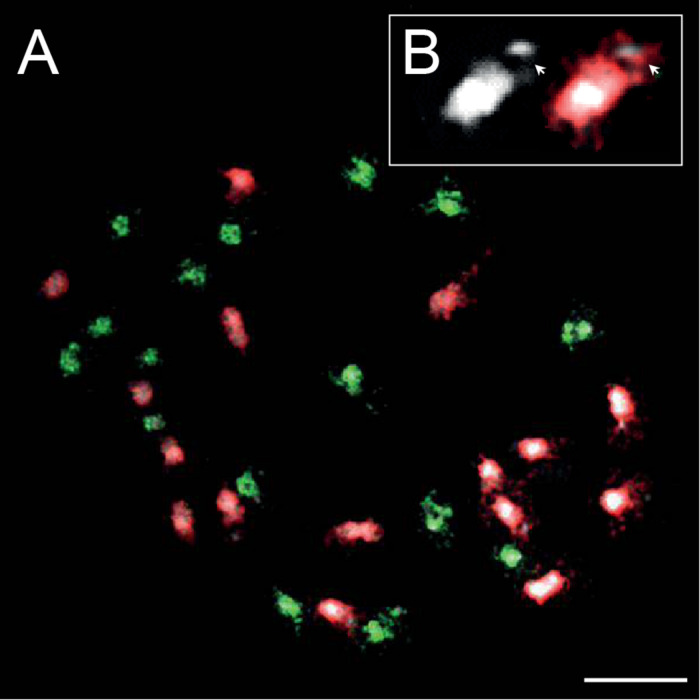
Genomic *in situ* hybridization (GISH) on mitotic chromosomes in the allotetraploid *Cardamine barbaraeoides* (2*n* = 32). **(A)** GISH with total genomic DNA of *Cardamine acris* subsp. *acris* (red fluorescence) and *Cardamine amara* subsp. *balcanica* (green fluorescence) revealed two subgenomes contributed by ancestors of the two diploid species. **(B)** Close-up view of a heterochromatic knob-bearing chromosome (the terminal knob marked by arrowheads). Chromosomes were counterstained by 4′,6-diamidino-2-phenylindole (DAPI); scale bar, 10 μm.

DAPI staining of mitotic chromosomes in *C. barbaraeoides* revealed 16 bigger (L) chromosomes with more extensive pericentromeric heterochromatin that were readily discernible from the other 16 smaller (S) chromosomes (Figure 8A). The L chromosomes carried terminal heterochromatin knobs (Figure 8B), which were previously reported in the *C. pratensis* group (Mandáková et al., 2013). The gDNA probes of the three tested accessions from the *C. amara* clade (*C. amara* subsp. *amara*, subsp. *balcanica*, and *C. lazica*) hybridized on 16S chromosomes of *C. barbaraeoides*; the signal strengths of all three probes were comparable. The gDNA probes of the other five accessions tested (*C. acris* subsp. *acris*, *C. matthioli*, *C. penzesii*, *C. rivularis*, and *C. uliginosa* from the Caucasus) hybridized on 16L chromosomes of *C. barbaraeoides*. Although quantification of hybridization signals is problematic, we observed stronger fluorescence of the gDNA probes of *C. acris* compared to the other probes tested (Figure 8A, Supplementary Figure 8). Thus, GISH data suggest that *C. barbaraeoides* is an allotetraploid that originated via hybridization between members or recent ancestors of the *C. amara* clade and the other major clade, where *C. acris* appeared to be the most likely parental candidate. These two major groups of species differ in their genome size (see above in *Chromosome Numbers and Genome Size Variation* and Figure 2). This difference is reflected by bigger chromosomes, more pericentromeric heterochromatin, and terminal heterochromatic knobs within the *C. acris*–like subgenome, in contrast to smaller chromosomes within the *C. amara*–like subgenome of *C. barbaraeoides* (Figure 8A).

## Discussion

### Evolutionary Relationships and Polyploid Speciation in Balkan *Cardamine*: Evidence From Phylogenomic and Cytogenetic Data

Uncovering phylogenetic relationships within recently diverged plant groups can be challenging even at the diploid level. Persistence of ancestral polymorphisms, low genetic divergence between species, and both past and contemporary interspecific gene flow hamper robust phylogenetic inferences (Naciri and Linder, 2015; see, e.g., Blanco-Pastor et al., 2012; Krak et al., 2013; Konowalik et al., 2015). In the *Cardamine* species complexes studied here, previously applied ITS and noncoding cpDNA Sanger sequences showed a low level of sequence polymorphism, as well as conflicting phylogenetic signal (Lihová et al., 2004a; Marhold et al., 2004). AFLP fingerprinting proved to be efficient at delimiting and describing species, in concordance with morphological, ploidy level, and distribution patterns (Lihová et al., 2003, 2004b), but performed poorly in phylogenetic inference (Marhold et al., 2004).

In the present study, we applied a target enrichment approach, recently shown to provide high resolution also at low phylogenetic levels between the closest relatives (Villaverde et al., 2018; Carter et al., 2019; Tomasello et al., 2020). Indeed, using custom, genus-specific probes, we were able to retrieve sequences from more than 1,000 nuclear genes from each sample. At the diploid level, the topologies of the ML tree obtained from concatenation of targeted loci and the coalescent-based species tree were fully congruent, which suggests a low degree of ILS, in accordance with high support in the species tree (Figure 3). Recently, it has been emphasized that allele phasing should be preferred to the use of consensus sequences (or “contig”, sensu Andermann et al., 2019) ignoring heterozygous positions and allelic variation, as it can improve phylogenetic inference and yield a more accurate tree estimate especially in recently diverged species (Andermann et al., 2019; Tomasello et al., 2020; but see Kates et al., 2018). Using either consensus or phased allele sequences, here we obtained the same species tree topology, which additionally supports the robustness of our data. Some topological conflicts, however, appeared between the nuclear- and plastome-derived phylogenetic trees (Figure 7). This plastid–nuclear discordance among the diploids, described in more detail below, can identify traces of interspecific gene flow and thus shed further light onto the evolutionary histories of *Cardamine* species. With more extensive sampling in the future, including all representatives of the studied species complex across Europe, this approach has great potential to infer their phylogeny comprehensively. Here we provide our first insights from the perspective of Balkan species.

In accordance with previous phylogenetic studies (Lihová et al., 2004a; Marhold et al., 2004; Carlsen et al., 2009), *C. amara* was supported here as a distinct phylogenetic lineage separated from the taxa classified within the other two species complexes (*C. pratensis* and *C. raphanifolia* groups). *C. amara* is a widespread and polymorphic Eurasian species consisting of several subspecies in Europe (Lihová et al., 2004a). *Cardamine lazica*, a species from the Pontic mountains and western Caucasus, was identified here as a sister species to *C. amara*, as already suggested by AFLPs, but not sufficiently resolved previously by Sanger sequence data (under the name *C. wiedemanniana*, Lihová et al., 2004a; Marhold et al., 2004). Furthermore, the present data supported the monophyly of *C. acris*, the most widely distributed Balkan endemic with extensive morphological and genetic variation and three subspecies recognized (Perný et al., 2004). One accession of *C. acris* (C015-107) was misplaced in the plastome tree (Supplementary Figure 7), which is most likely a sign of interspecific hybridization or introgression, a scenario supported also by a mixture of divergent nrDNA variants found in this individual (Supplementary Figure 6). Furthermore, both nuclear and plastid data congruently revealed a sister species relationship between *C. acris* and the population from the Uludağ Mts. in northwestern Turkey, classified as *C.* cf. *uliginosa*. On the other hand, the population of *C. uliginosa* from the Caucasus was genetically divergent. *C. uliginosa* grows across Anatolia (but with very scarce records from its western parts except for the Uludağ Mts., J. Kučera, pers. comm.) and the Caucasus, extending further to the south and south-east, reaching the mountains of Iran and Lebanon. Previous studies have already indicated that it is a highly polymorphic species (Marhold et al., 2004), maybe even a complex of (cryptic) species.

The relationships between the other four species, *C. apennina*, *C. matthioli*, *C. penzesii*, and *C. rivularis*, all traditionally classified within the *C. pratensis* group, showed conflicting patterns between nuclear and plastid trees but also low support (Figure 7). Both ongoing and past gene flows, the latter probably facilitated by range shifts in glacial-interglacial periods, have been inferred to occur between *C. matthioli* and *C. rivularis* in Bulgaria (Ančev et al., 2013; Melichárková et al., 2020), which may explain their close position in the plastid tree, in contrast to the nuclear tree. The lowland species *C. penzesii* was resolved as sister to *C. uliginosa* from the Caucasus in nuclear trees, whereas the positions of both species remained uncertain in plastome trees. PhyloNet analyses of nuclear loci, which account for both ILS and interspecific gene flow (Wen et al., 2018), as well as the presence of divergent nrDNA variants, suggested a reticulate evolutionary history of *C. penzesii* (Supplementary Figures 2, 6).

Our Hyb-Seq and GISH results provide strong evidence that *C. barbaraeoides*, a stenoendemic of the Southern Pindos Mts., is of allotetraploid origin. Interestingly, the phylogenetic placements of its homeologs do not favor a very recent (i.e., postglacial) origin, as might have been suspected from its narrow range within the area occupied by *C. acris*. Alleles retrieved from two subgenomes appeared differentiated from those observed in present-day diploids, suggesting that the parental species of *C. barbaraeoides* were most likely the common ancestors of *C. amara* and *C. lazica* on one side (the maternal one, as inferred from cpDNA) and of *C. acris* and a western Anatolian taxon (so far attributed to *C. uliginosa*) on the other (Figures 5, 7). A possible alternative scenario is that extensive genomic changes in the polyploid in response to a “genomic shock,” including nonhomologous recombination, have significantly altered and differentiated the polyploid genome from its diploid progenitors (Nieto Feliner and Rosselló, 2012; Madlung and Wendel, 2013). Still, the former hypothesis of an older allopolyploidization event and a relict character of this species is favored also by the plastome tree, which confirmed the same phylogenetic placement of *C. barbaraeoides* as was revealed for the “A” homeolog in the nuclear species trees. The phylogenetic patterns also agreed with the strength of GISH signal, where both *C. amara* and *C. lazica* probes hybridized well on S chromosomes of *C. barbaraeoides*, whereas *C. acris* probes hybridized stronger on L chromosomes (Uludağ accessions were not available) than the other diploids analyzed (Supplementary Figure 8).

Based on a recently published tribe-wide dated phylogeny (Huang et al., 2020), we can infer early- to mid-Pleistocene divergence among the diploids analyzed here within both major clades, which suggests also the approximate age of this allopolyploid. The highly restricted occurrence of *C. barbaraeoides* at only a few sites within the small range of the Lakmos Mts. (S Pindos) remains intriguing. We may speculate whether the present occurrence is only a remnant of a previously wider range, or whether the allopolyploidization event took place within the current area and the species never expanded much beyond it. The former hypothesis appears more plausible when we reject the species’ very recent origin and consider also evidence that Mediterranean mountains have experienced significant changes in vegetation, habitat availability, and diversity during Quaternary climatic oscillations (Médail and Diadema, 2009; Nieto Feliner, 2014).

### Drivers of Speciation Within the *Cardamine* Species Complexes: The Role of Mountains of the Balkan Peninsula and Adjacent Biogeographic Regions in Shaping Diversity and Endemism Patterns

The *Cardamine* taxa under study exhibit parapatric to allopatric distributions, and all occupy similar wet habitats, partly with different elevational preferences (Figure 1). From the presented phylogenetic reconstructions, we can infer that they likely evolved via both allopatric and ecological speciation processes, which have also been affected by interspecific gene flow. The studied species complexes comprise numerous endemics not only in the Balkan Peninsula but also across the other parts of the Mediterranean (Marhold et al., 2018). The prevalence of endemics in the Mediterranean, along with the commonly observed geographically structured genetic variation within several species (Lihová et al., 2003; Perný et al., 2005a, b; Melichárková et al., 2020), suggests that geographic isolation played an important evolutionary role. These patterns may reflect past range fragmentation in response to Pleistocene climatic oscillations (Nieto Feliner, 2014), as well as spatially restricted gene flow and species dispersal, which may be the two principal causes, acting in concert, of the current high endemism rate in these species complexes. Lowland-alpine species pairs, such as *C. penzesii* and *C. uliginosa*, also show signs of ecological speciation as a result of adaptation to habitats at high elevations, typically with higher precipitation and solar radiation input, and lower temperatures, as proven for *C. amara* subsp. *amara* and subsp. *austriaca* in the Eastern Alps (Zozomová-Lihová et al., 2015). Ecological niche analyses in four species of the *C. pratensis* complex growing from lowlands up to the alpine belt in Central and Southeastern Europe (including *C. rivularis* and *C. matthioli* studied here; Melichárková et al., 2020) found niche shifts and niche breadth differences, but still considerable niche overlaps among species, representing both sympatric and allopatric cases. It appears that divergent ecological requirements may play a certain role in the evolution of these species complexes but probably do not constitute a strong constraint that would significantly hamper range expansion and explain the high incidence of endemics.

With the present results, we provide additional support for the prominent role of Mediterranean mountains both as cradles and reservoirs of species and genetic diversity and, more specifically, for the contribution of polyploid speciation to the origin of biodiversity hotspots. Indeed, the Southern Pindos range, the area of *C. barbaraeoides*, is recognized as an important center of endemism and also a refugial area (Stevanović et al., 2007; Médail and Diadema, 2009; Kougioumoutzis et al., 2021). Quaternary climatic oscillations have led to species range shifts, repeated range fragmentation, and reduction followed by expansion, and these processes have facilitated contacts between previously isolated lineages and brought opportunities for hybridization (Nieto Feliner, 2014; Marques et al., 2018; see, e.g., Blanco-Pastor et al., 2012; Maguilla et al., 2017; Zozomová-Lihová et al., 2020). The great ecological and topographic heterogeneity of Mediterranean mountains has likely favored not only hybridization events, but also the establishment and persistence of newly formed allopolyploids. Several examples of polyploid endemics confined to some mountains of the Balkan Peninsula (e.g., Cires et al., 2014; Olšavská et al., 2016; Španiel et al., 2017; López-González et al., 2021) suggest that allopolyploid speciation may significantly contribute to the diversity of the Balkan endemic mountain flora, but this topic is still poorly explored, and further studies are needed.

Our present study revealed cases in which Balkan taxa have their phylogenetically closest counterparts in the Anatolian or Caucasus regions, in support of the known biogeographic links between these areas (Strid, 1986; Bilgin, 2011; Thompson, 2020). Indeed, the Anatolian phytogeographic element is well represented in the Greek mountain flora, and this is particularly true for species distributed in the Uludağ Mts. in northwestern Turkey (Strid, 1986). The Aegean Sea, the Sea of Marmara, and the Thracian Plain are significant barriers to mountain species dispersal between the Balkan Peninsula and Anatolia at present (Ansell et al., 2011; Bilgin, 2011). However, they may have been penetrated especially in colder periods at the Pliocene–Pleistocene transition and during Pleistocene glaciations (Strid, 1986; Ansell et al., 2011). One of the common phylogeographic patterns recognized in Anatolia suggests a genetic break within Anatolia, differentiating populations in western Anatolia and the Balkan Peninsula from those in eastern Anatolia (Bilgin, 2011). This pattern resembles the present case of high affinity between *C. acris* and the population from the Uludağ Mts.; however, more detailed studies of *C. uliginosa* across its distribution range are needed. Furthermore, closer evolutionary relationships and traces of hybridization between *C. penzesii* from flood-plain forests near the Black Sea coast and high-mountain *C. uliginosa* from the Caucasus seem to support the Northern Anatolian dispersal corridor (Kaya and Çiplak, 2017; Özüdoğru and Mummenhoff, 2020). Northern Anatolia may have provided sites ecologically suitable for both lowland and mountain population survival in close proximity and allowed for allopatric, as well as ecological speciation (Kučera et al., 2006; Roces-Díaz et al., 2018).

### Resolving Allopolyploid Origins From Hyb-Seq Data and Potential of nrDNA Polymorphisms for Detecting Reticulate Evolution

The employment of low-copy nuclear genes in phylogenetic studies, especially when polyploids are involved, is crucial. Nuclear genes show biparental inheritance and typically retain evidence of a reticulate history (e.g., Brysting et al., 2007; Rousseau-Gueutin et al., 2009; Brassac and Blattner, 2015). Still, it is known that individual gene trees may show discordant histories that do not match the true evolutionary history, because of various processes related to the complexity of nuclear genomes, such as high allelic variation and ILS, nonhomologous recombination, gene duplication, and gene loss (Maddison, 1997; Small et al., 2004; Degnan and Rosenberg, 2009). Therefore, the use of multiple unlinked loci has been strongly advised (Naciri and Linder, 2015). Target enrichment techniques that may capture hundreds of unlinked orthologous loci are promising in resolving the origins and evolutionary histories of polyploid species with much greater confidence (Kamneva et al., 2017). Assembly of short sequence reads, however, remains a challenge for allopolyploid genomes, because a mixture of reads belonging to both homologous and homeologous loci is obtained (Kyriakidou et al., 2018). Most phylogenetic studies have used consensus assembly (e.g., Crowl et al., 2017; Morales-Briones et al., 2018; Carter et al., 2019), that is, a single majority sequence at a given locus. This means, however, that sequences from different homeologs (parental subgenomes), as well as chimeric sequences, may be retrieved. Allopolyploid speciation is then commonly inferred by network analyses, which account for both ILS and hybridization (Crowl et al., 2017; Morales-Briones et al., 2018; Carter et al., 2019).

In the present study, we employed the network analyses based on the consensus sequences, which, in congruence with the SNP data analyses, identified conflicting signal within the data and suggested allotetraploid origin of *C. barbaraeoides*. Nevertheless, as a significant step further, we proceeded to allele assembly and sorting. Some approaches or tools for assembling allele sequences and distinguishing among homeologs have recently been proposed for polyploids (Page et al., 2013; Kamneva et al., 2017; Rothfels et al., 2017; Schrinner et al., 2020; Rothfels, 2021). Several previous studies used parallel amplicon sequencing to analyze polyploid species, but capturing only a low number of loci (up to 12 loci) and with manual homeolog identification and sorting (Brassac and Blattner, 2015; Rothfels et al., 2017; Dauphin et al., 2018; see also Eriksson et al., 2018, specifically for target enrichment data). Here we propose a novel approach in which hundreds of loci obtained from target enrichment techniques can be analyzed simultaneously and allele sorting does not require manual inspection and labeling. We inferred phased alleles based on available tools and developed a bioinformatics procedure to sort them into homeologs. Allele sorting is based on calculating distances between alleles, obtained from branch lengths of corresponding gene trees, first between alleles from a given polyploid (to identify allele pairs) and then from its diploid relatives. Homeolog labeling is based on allele pair distances to the suspected maternal species, as identified by plastome analyses. The phylogenetic positions of the obtained homeologs, representing two parental subgenomes in the polyploid, are then explored by a species tree inference. This approach is most straightforward when the maternal species is at least approximately determined, but could be applied even if this information is unknown, in the case of missing cpDNA data, a possibly extinct or an unsampled maternal parent. Under such scenarios, one of the most closely related species, a possible progenitor of the investigated polyploid, could be identified from the network analyses inferred from the consensus sequences and subsequently used for homeolog labeling.

Two shortcomings may potentially limit the efficiency of our approach. One is specific to the target loci and/or species studied. Successful allele sorting in polyploids, namely, depends on both parental genome divergence and the informativeness (phylogenetic signal) of target loci. Alleles from some genes may not be unequivocally sorted into homeologs, because of low phylogenetic signal and low sequence divergence. Still, when employing a large set of target loci during sequence capture and including also more variable flanking intronic and intergenic regions (as is achieved via the Hyb-Seq approach; Weitemier et al., 2014), sufficient data and resolution can be obtained. Here we demonstrate that with several reduced datasets, allowing either missing accessions or loci, we obtained the same topologies of the species trees, and the same allopolyploid scenario was inferred. The second obstacle is related to the short length of sequence reads obtained from the Illumina platform, which throws down a challenge to allele phasing software. The shorter length of sequence reads more often causes sequence splitting into multiple phase blocks. Variant sites are phased with other sites within the given block but cannot be phased with respect to variants in the other blocks because of insufficient read data between the blocks (see Kates et al., 2018). If multiple phase blocks occurred, phased alleles were retained only in the largest phase block, and the remaining intraindividual variants were masked (12.92% of SNPs per sample in average). Concatenation of exon sequences to genes has a dual (partially contradicting) effect. The sequence length has a positive effect on the resolution of the phylogenetic tree. On the other hand, concatenation involved both sorted and unsorted (with masked interallelic SNPs) exons, which means that interallelic variation was partly homogenized. To investigate the impact of this issue on phylogenetic reconstruction, we compared two datasets that differed in the length of the loci used and the amount of masked SNP variants: shorter exon-based and longer gene-based datasets. Only a single topological difference was observed between the species trees inferred from these datasets, inspected in more detail by running GGI topology tests (Arcila et al., 2017) and suggesting that this issue may be worth considering. Overall, we demonstrate that allele phasing and distinguishing homeologous copies are crucial for determining the origin of polyploids and for resolving reticulate evolution of polyploid complexes. The here proposed approach works so far for suspected allotetraploids, but future developments will focus on resolving genomes of higher ploidy levels that may be composed of more than two subgenomes (such as *Cardamine occulta* and *Cardamine schulzii*, both identified as trigenomic allopolyploids by advanced cytogenetic techniques; Mandáková et al., 2013, 2019), as well as autopolyploids.

Genome skimming, performed as part of the Hyb-Seq approach (Weitemier et al., 2014), allowed us to assemble also the high-copy nrDNA with sufficient coverage and to compare it with the variation obtained by molecular cloning. Molecular cloning found substantial intragenomic variation in most species studied, in agreement with the commonly observed patterns that concerted evolution acting in nrDNA may be incomplete (Álvarez and Wendel, 2003; Weitemier et al., 2015). Because high-throughput sequencing recovers reads from all potential repeat variants within and among nrDNA loci, we explored different possibilities how to deal with such intraindividual polymorphisms. We compared the two most commonly used coding schemes for such polymorphisms, the consensus (majority) one and the ambiguous one (Vargas et al., 2017; Fonseca and Lohmann, 2019), with phasing that enables to extract different sequence variants comparable to those obtained through cloning. Indeed, as we revealed in the cases of *C. penzesii* and *C. acris*, phasing may be an efficient way to recover phylogenetically relevant intragenomic nrDNA variation, suggesting a reticulate history, which replaces laborious cloning and PCR amplifications. On the other hand, really rare variants, such as those in *C. barbaraeoides* that have apparently remained as traces from its paternal progenitor, may be difficult to obtain from genome skim data and require improvements in bioinformatics tools. By contrast, with the amplicon sequencing approach, Tkach et al. (2019) were able to detect extremely rare (present in as few as 0.2% reads) ITS2 variants that indicated ancient hybridization events. Therefore, although genome skim data are easy to obtain and provide huge amounts of data from both organellar and nuclear DNA high-copy fractions, they should be considered with caution especially in groups with reticulate evolutionary histories (e.g., Vargas et al., 2017; del Valle et al., 2019; Chen et al., 2020). With the recently increasing efforts to develop target enrichment probes specific to relatively narrow focus groups (e.g., Schmickl et al., 2016; Vatanparast et al., 2018; Nikolov et al., 2019), this approach will become available for a wider spectrum of taxa, and genome skimming may become a useful complement to, phylogenetically more robust, datasets of hundreds of independent nuclear loci.

## Conclusion

Our study demonstrates the importance of a thorough phylogenomic approach when studying the evolution of recently diverged species complexes affected by reticulation events at both the diploid and polyploid levels. We emphasize the significance of retrieving allelic and homeologous variation from nuclear genes, as well as divergent nrDNA copy variants from high-throughput genomic data. Along with the employment of multiple analysis methods, they all, in concert, allow to resolve the origins of polyploids, detect cases of interspecific gene flow, and explain plastid–nuclear phylogenetic discordance. We suggest that despite recent advances in phylogenomic data analyses, significant improvements are needed especially in processing and analyzing sequence data from polyploid and hybrid genomes. With the present results, we also illustrate the prominent role of Mediterranean mountains as biodiversity hotspots, favoring long-term survival and speciation in allopatry, but also acting as melting pots that promote secondary contacts between species, hybridization, and polyploid evolution.

## Data Availability Statement

The datasets presented in this study can be found in online repositories. The names of the repository/repositories and accession number(s) can be found below: https://www.ncbi.nlm.nih.gov/genbank/, PRJNA687126; https://www.ncbi.nlm.nih.gov/genbank/, MW476310–MW476485, MW480861–MW480862, and MW435615–MW435620.

## Author Contributions

JZ-L, KM, and MŠe conceived and designed study. MŠe, MP, KM, MSo, AK, and JZ-L collected plant material. AK, JZ-L, TM, MC, and MP performed laboratory work. AK, MŠe, JZ-L, and TM analyzed the data. MŠe performed bioinformatics scripting. RS contributed to bait development and Hyb-Seq protocol optimization. JZ-L and MŠe wrote the manuscript with contributions from KM and MSo. All authors have discussed, read, and commented on the manuscript.

## Conflict of Interest

The authors declare that the research was conducted in the absence of any commercial or financial relationships that could be construed as a potential conflict of interest.

## Footnotes

1 https://github.com/V-Z/sondovac

2 https://jgi.doe.gov/data-and-tools/bbtools
