# Supplementary material for: Allele Sorting as a Novel Approach to Resolving the Origin of Allotetraploids Using Hyb-Seq Data: A Case Study of the Balkan Mountain Endemic Cardamine barbaraeoides
Source: Front Plant Sci. 2021 Apr 28;12:659275. doi: 10.3389/fpls.2021.659275 (PMC8115912; doi:10.3389/fpls.2021.659275)

Supplementary Figure 1. Maximum likelihood tree inferred in RAxML-NG from concatenated sequences of all 1,168 target nuclear genes in diploid *Cardamine* accessions, based on consensus supercontig sequences. Branch support is shown with bootstrap values and quartet sampling scores (QC/QD/QI); in addition, coloured circles in the nodes indicate QC value intervals.

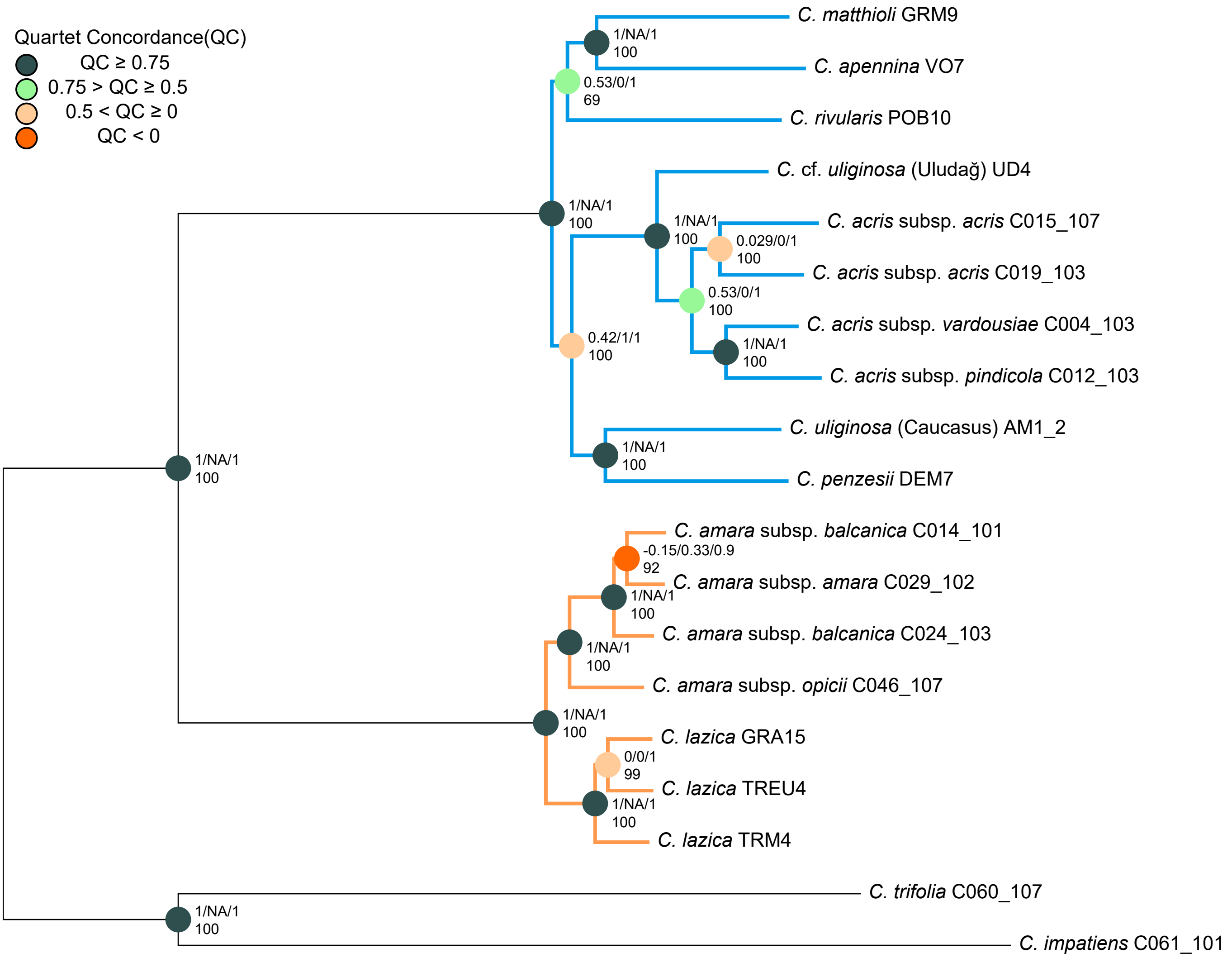

Supplementary Figure 2. Species network inferred in PhyloNet from the set of 1,168 nuclear gene trees indicating reticulated origin of *Cardamine penzesii*. Inheritance probabilities are shown along the bra

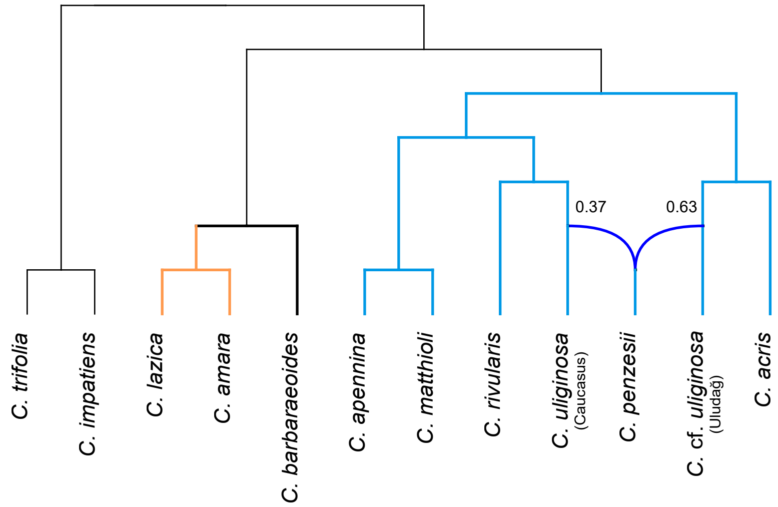

Supplementary Figure 3. Pie charts depicting the proportion of homozygous and heterozygous exons recovered in *Cardamine* samples by read-backed phasing of 1,829 targeted exons. For details on the accession labels, see **Supplementary Data 1**.

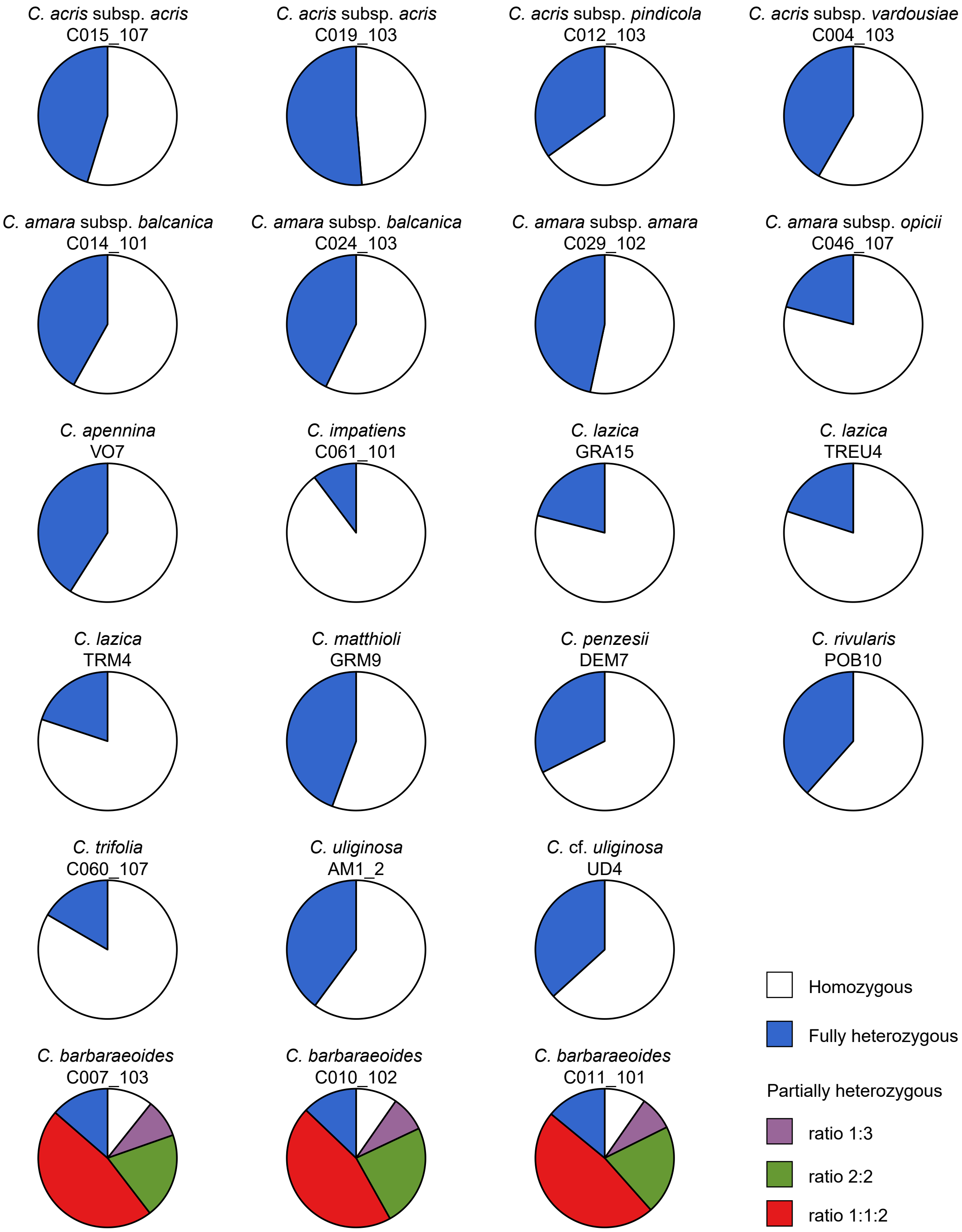

Supplementary Figure 4. Species trees inferred in ASTRAL-III based on phased allele sequences, with alleles of the tetraploid *Cardamine barbaraeoides* sorted into A and B homeologs. Either the alignments of exon (supercontig) sequences were used and ML exon trees were inferred, or the exons were concatenated to genes, and ML gene trees were constructed. Allele sorting was equivocal for some accessions in some exons/genes; therefore, several datasets were assembled with the following resulting species trees: (**A**) a selection of 612 exons with all three accessions of *C. barbaraeoides* retained; (**B**) a selection of 974 exons with at least two accessions of *C. barbaraeoides* retained; (**C**) all 1,829 exons with zero to three accessions of *C. barbaraeoides* per exon. (**D**) a selection of 274 genes with all three accessions of *C. barbaraeoides* retained; (**E**) a selection of 441 genes with at least two accessions of *C. barbaraeoides* retained; (**F**) all 1,168 genes with zero to three accessions of *C. barbaraeoides* per gene. Branch support is indicated by pie-charts, depicting three local posterior probabilities for the given branch (dark blue for the main topology as resolved here and light blue for the alternative ones). Below each species tree, also graphs depicting the results of gene genealogy interrogation analyses (GGI) are shown. The GGI analyses tested three different phylogenetic placements of the homeologs identified in the tetraploid *C. barbaraeoides*, as indicated and depicted in different colours. For **A**, **B**, **D** and **E**, the GGI analyses were based on the same sets of genes or exons as the corresponding species trees. In (**C**) a dataset of 1,287 exons, for which alleles from at least one accession of *C. barbaraeoides* (i.e. with one to three accessions per exon) were successfully phased and sorted, was used. In (**F**) a dataset of 621 genes, for which alleles from at least one accession of *C. barbaraeoides* were successfully phased and sorted, was used. The plots show the cumulative number of constrained gene trees which support the given topology and their P values obtained from approximately unbiased (AU) tests. Curves above the dashed lines indicate the number of trees that support the given topology significantly better (P≤0.05) than the alternative ones.

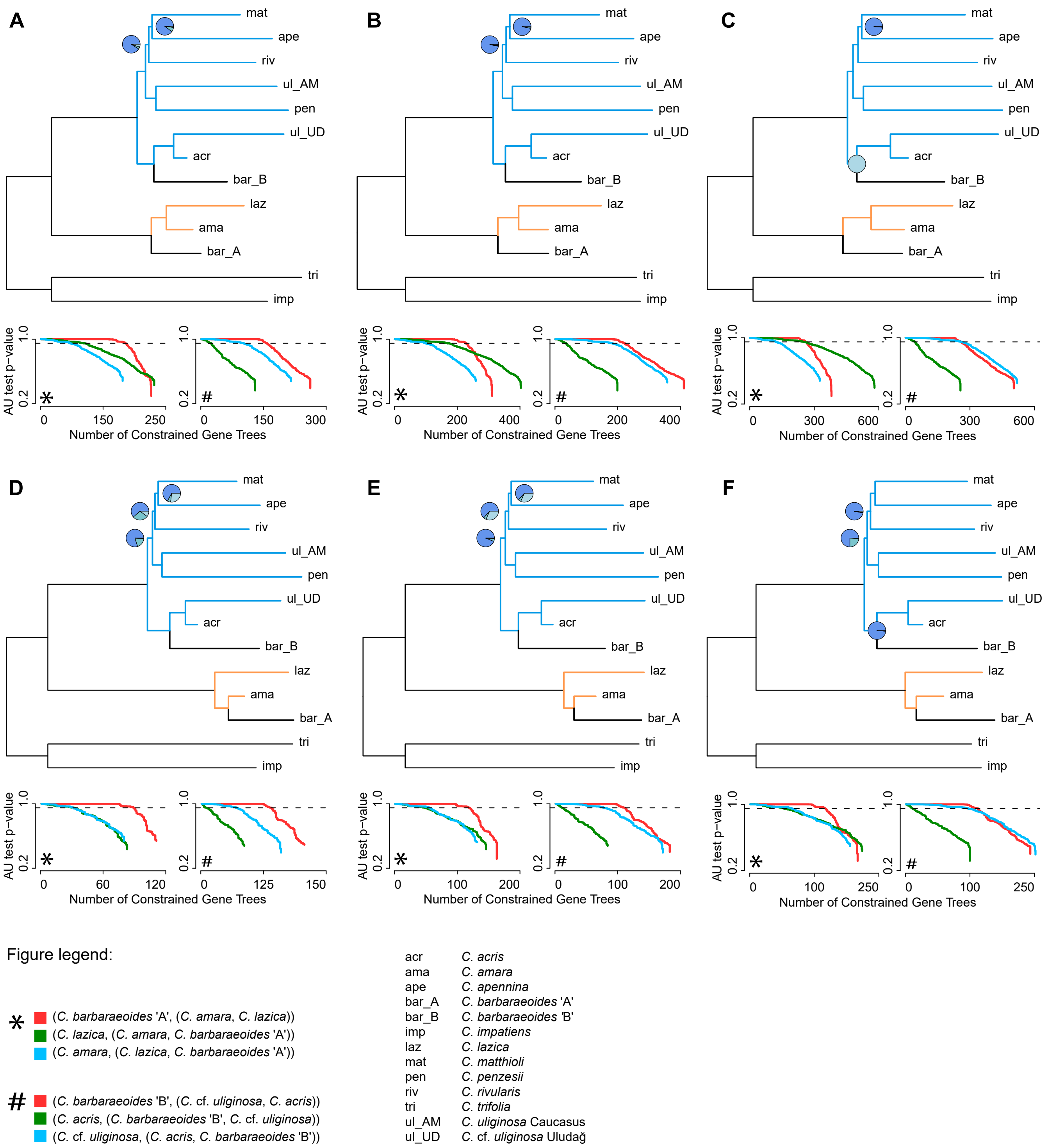

Supplementary Figure 5. Boxplots graphs depicting genetic distances between the phased alleles of the tetraploid *Cardamine barbaraeoides*, sorted into A and B homeologs, and the alleles of the diploids. The presented allelic distances were calculated as the sum of branch lengths of the corresponding exon or gene ML trees. The datasets of 612 exons or 274 genes were employed here, for which the alleles of all three accessions of *C. barbaraeoides* were successfully phased and sorted into A and B homeologs.

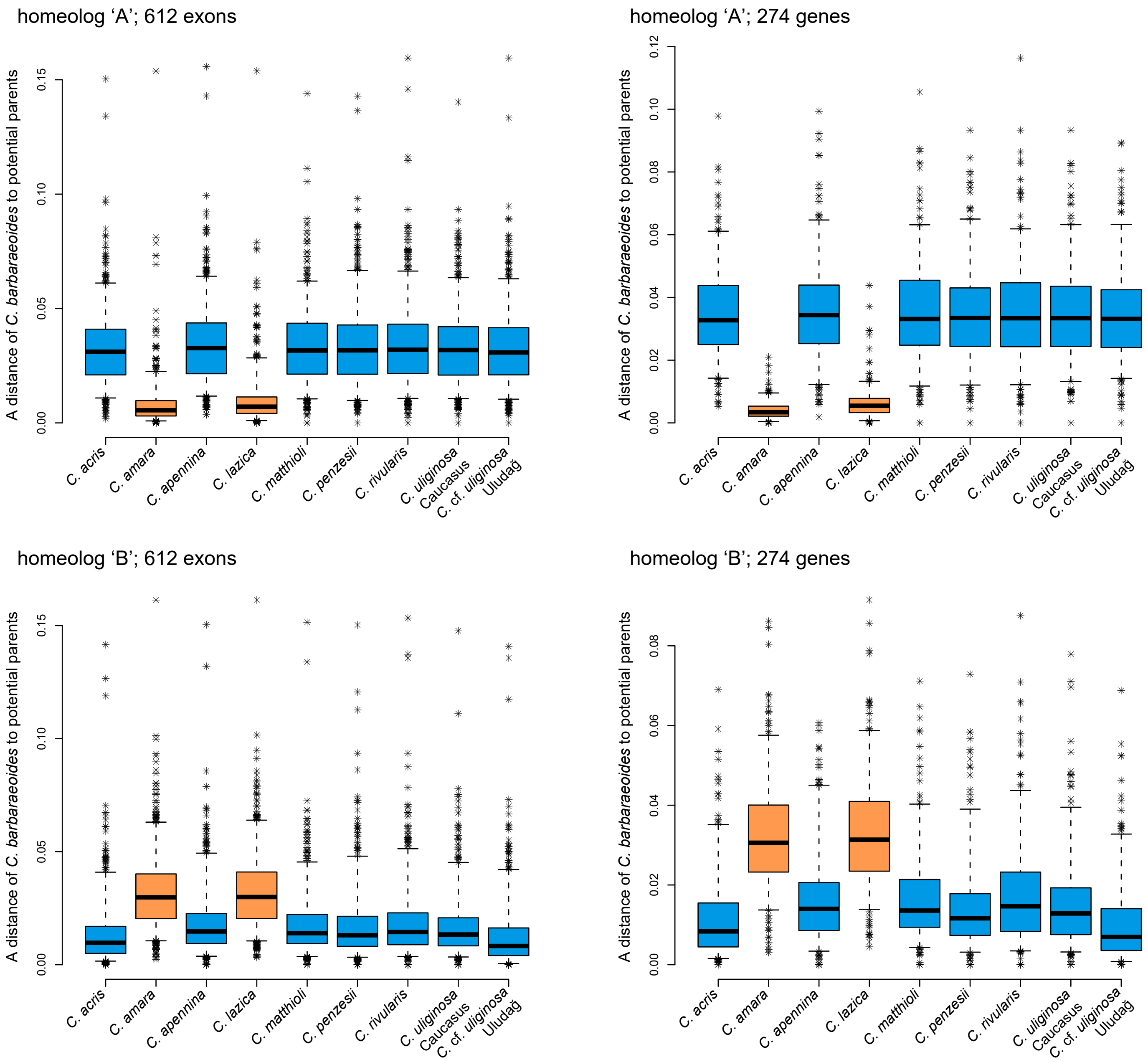

Supplementary Figure 6. Maximum likelihood trees inferred in RAxML from nrDNA (ITS1-5.8S-ITS2 region) sequences obtained from molecular cloning and Sanger sequencing (**cloning**) and from genome skim data from Hyb-Seq: ‘multiallelic’ dataset from read-backed phasing (**phasing**), ambiguous assembly with intraindividual SNPs replaced by IUPAC codes (**ambiguity**), and consensus assembly following the majority rule criterion (**consensus**). Positions of sequences obtained from *C. barbaraeoides* (green), *C. penzesii* (light brown), and one divergent sequence of *C. acris* (red) are highligted. Molecular cloning was based on an extended sampling of 48 individuals whereas the Hyb-Seq data comprised 20 ingroup individuals. Values above branches are bootstrap support >50%. For details on the accession labels, see **Supplementary Data 1.**

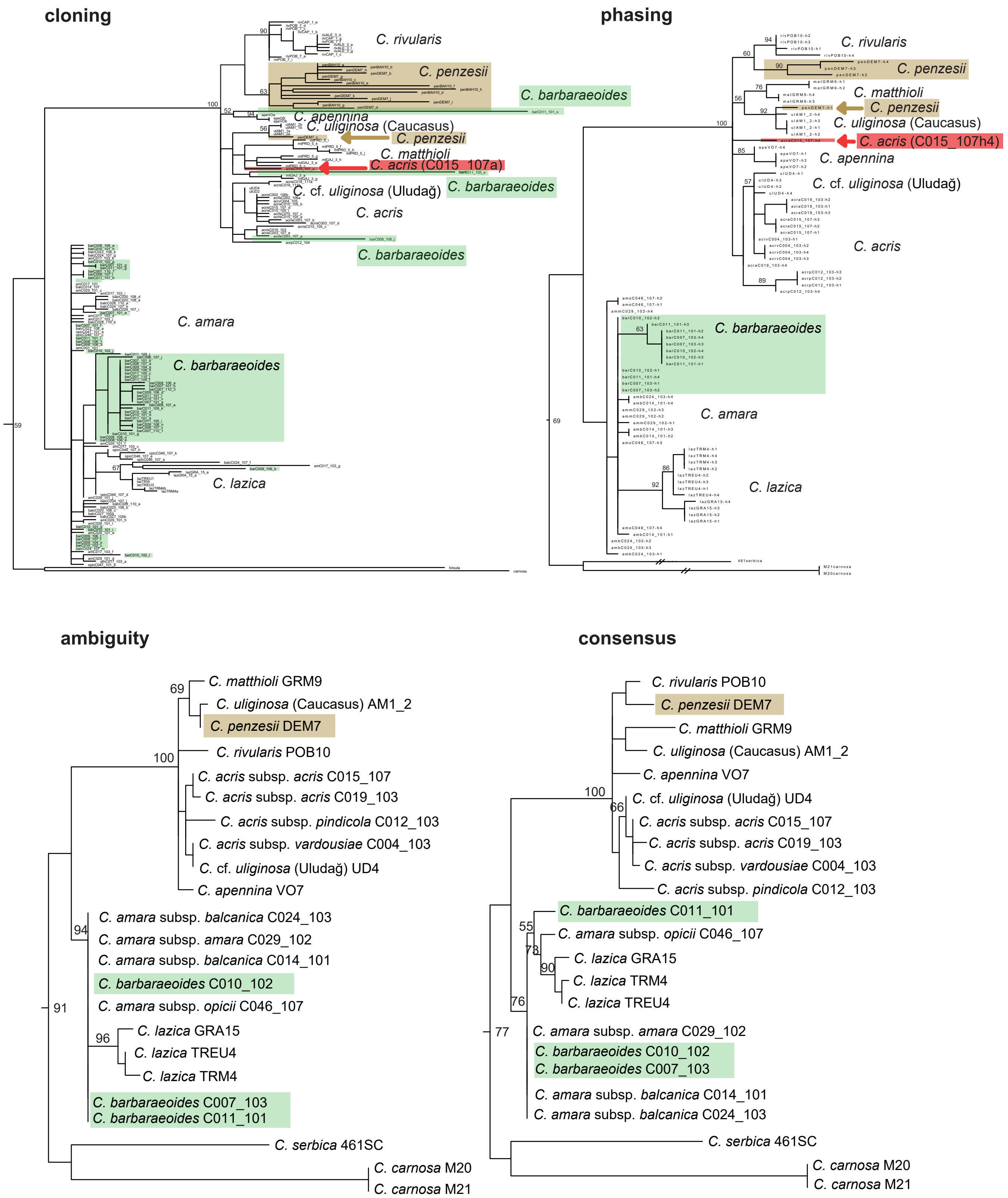

Supplementary Figure 7. Maximum likelihood trees of complete chloroplast (LSC, SSC, IRb) sequences (**A**) or concatenated chloroplast genes (protein-coding, rRNA and tRNA genes), omitting intergenic spacers (**B**), both inferred in RAxML. Bootstrap values are shown above branches. For details on the accession labels, see **Supplementary Data 1**.

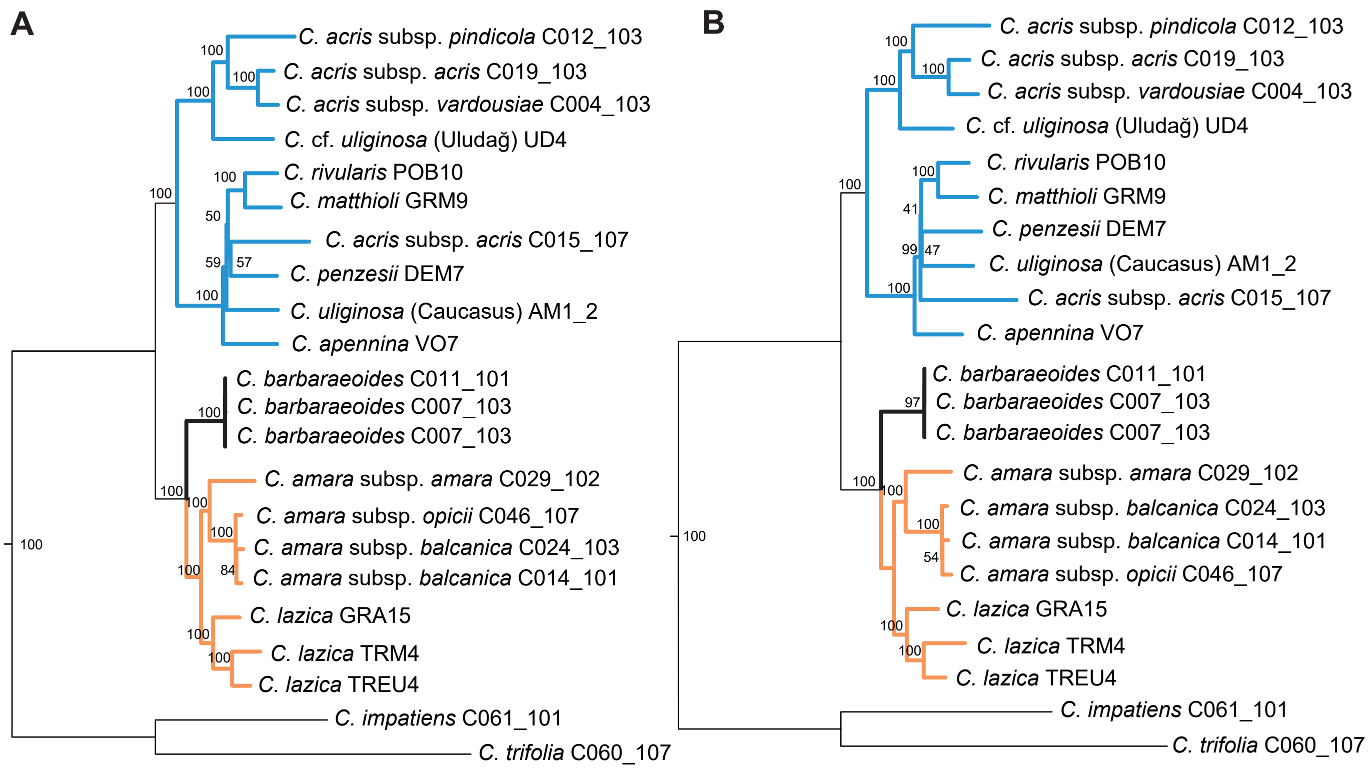

Supplementary Figure 8. Genomic *in situ* hybridization (GISH) on mitotic chromosomes in the allotetraploid *Cardamine barbaraeoides* (2n = 32). GISH with different combinations of total genomic DNA of *C. amara* subsp. *amara* (ama), *C. amara* subsp. *balcanica* (amb), *C. lazica* (laz), *C. acris* subsp. *acris* (acr), *C. matthioli* (mat), *C. rivularis* (riv) and *C. penzesii* (pen) revealed two subgenomes contributed by ancestors of the diploid species. Chromosomes were counterstained by 4', 6-diamidino-2-phenylindole (DAPI); scale bars – 10 µm.

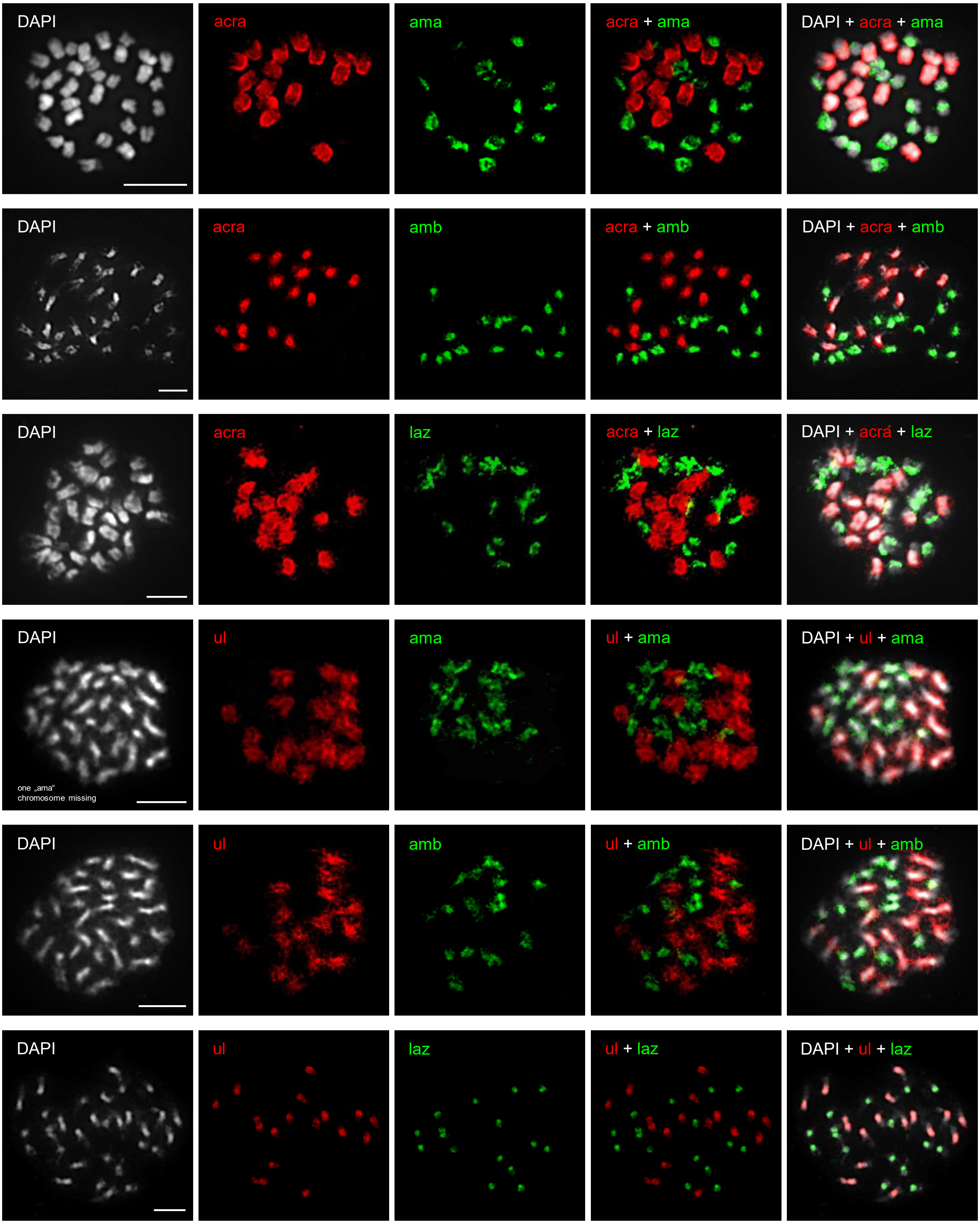

Supplementary Figure 8 (cont.). Genomic *in situ* hybridization (GISH) on mitotic chromosomes in the allotetraploid *Cardamine barbaraeoides* (2n = 32). GISH with different combinations of total genomic DNA of *C. amara* subsp. *amara* (ama), *C. amara* subsp. *balcanica* (amb), *C. lazica* (laz), *C. acris* subsp. *acris* (acr), *C. matthioli* (mat), *C. rivularis* (riv) and *C. penzesii* (pen) revealed two subgenomes contributed by ancestors of the diploid species. Chromosomes were counter stained by 4’, 6-diamidino-2-phenylindole (DAPI); scale bars – 10 µm.

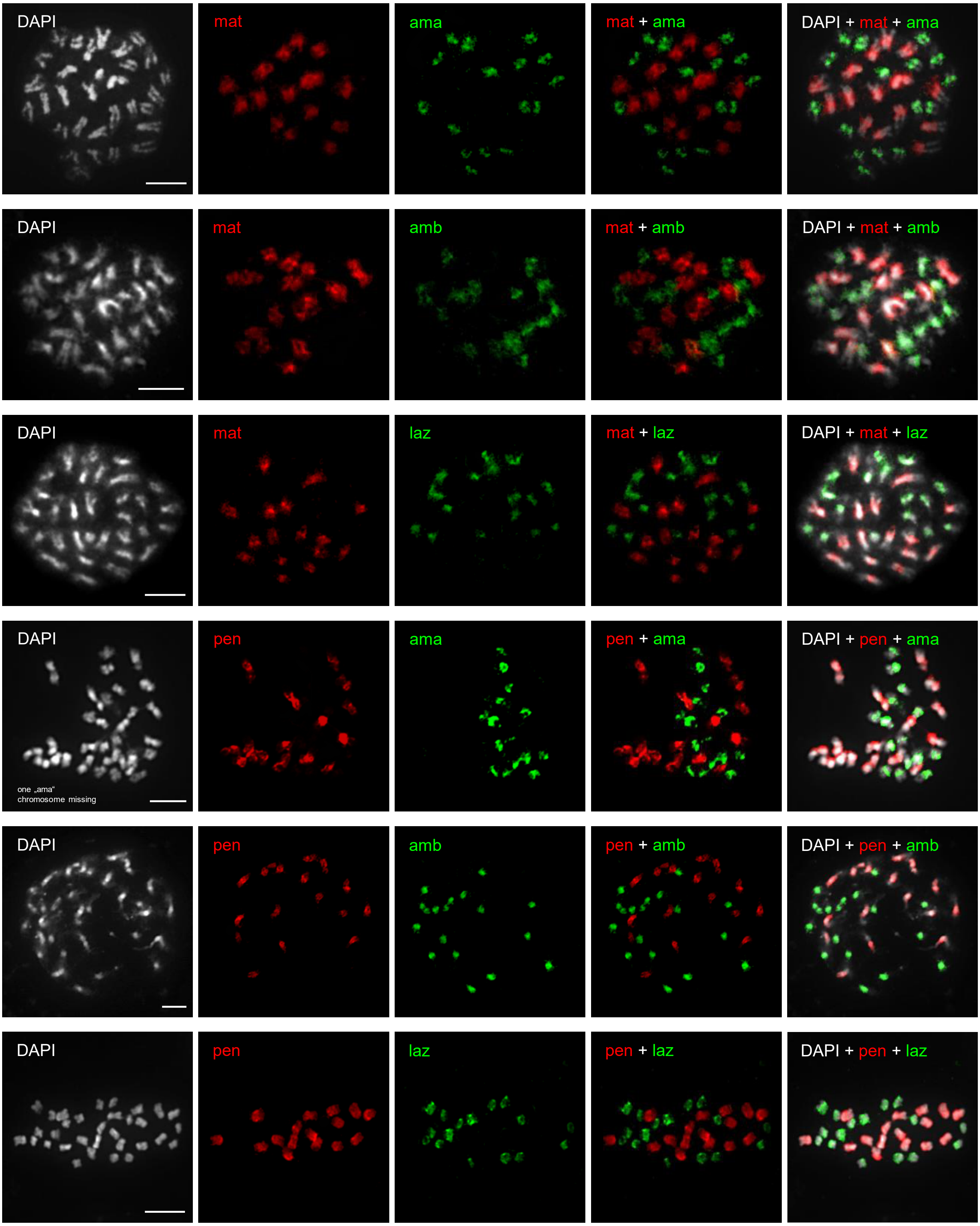

Supplementary Figure 8 (cont.). Genomic *in situ* hybridization (GISH) on mitotic chromosomes in the allotetraploid *Cardamine barbaraeoides* (2n = 32). GISH with different combinations of total genomic DNA of *C. amara* subsp. *amara* (ama), *C. amara* subsp. *balcanica* (amb), *C. lazica* (laz), *C. acris* subsp. *acris* (acr), *C. matthioli* (mat), *C. rivularis* (riv) and *C. penzesii* (pen) revealed two subgenomes contributed by ancestors of the diploid species. Chromosomes were counter stained by 4’, 6-diamidino-2-phenylindole (DAPI); scale bars – 10 µm.

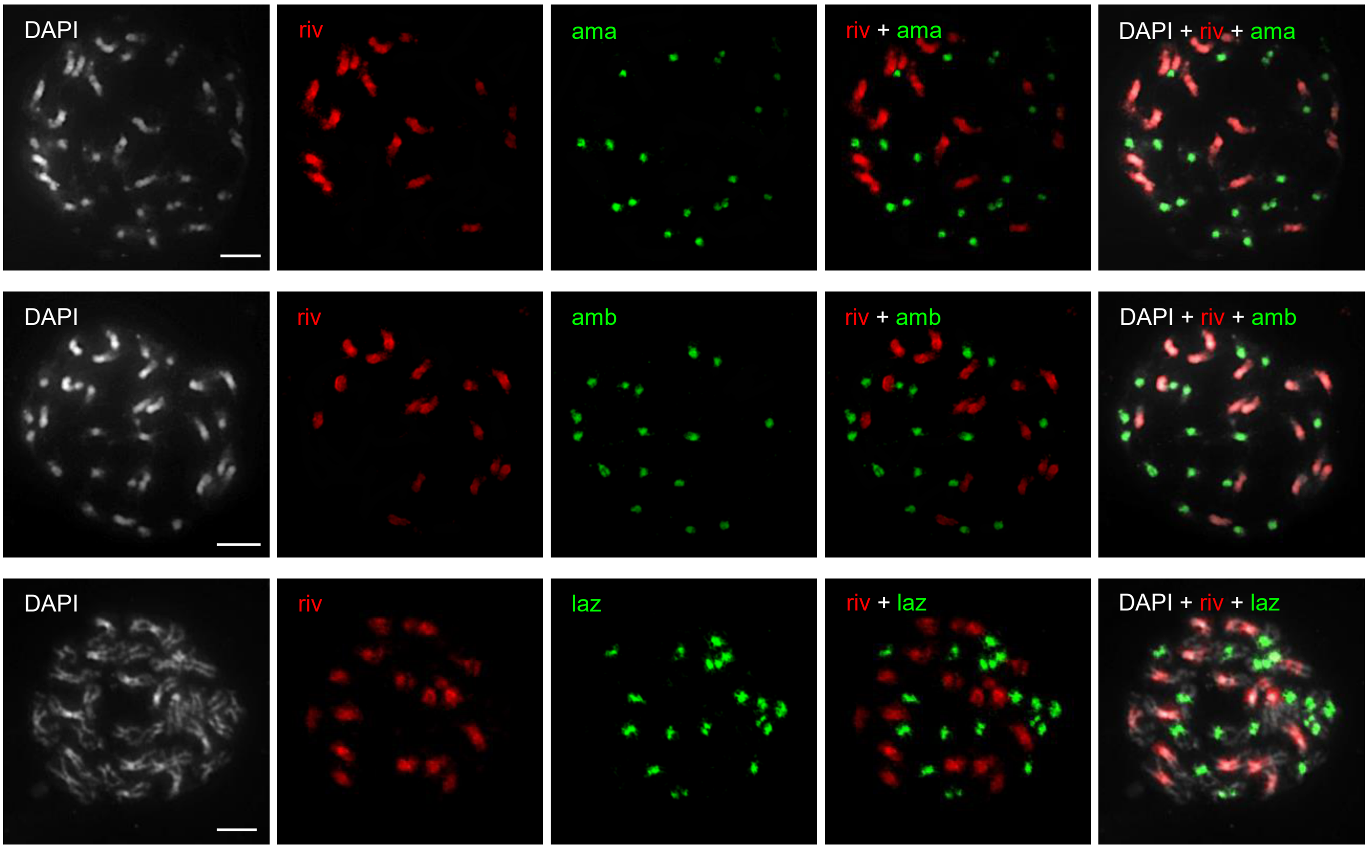

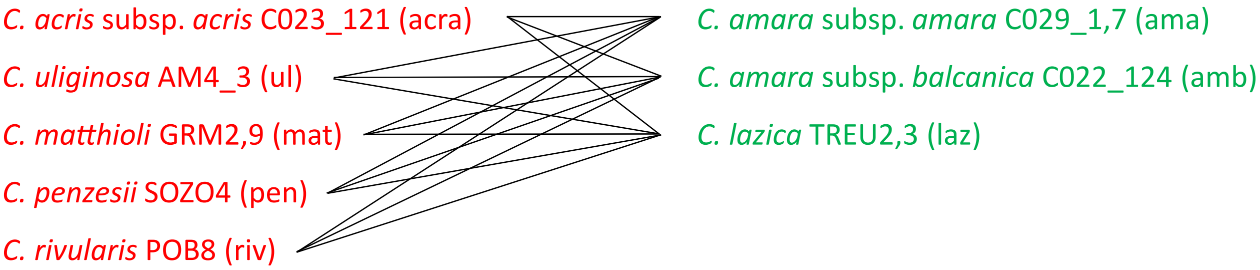

Supplement: Supplementary file 1 [file Data_Sheet_1.zip › Supplementary Figures 1-8.pdf]
