# Supplementary material for: Allele Sorting as a Novel Approach to Resolving the Origin of Allotetraploids Using Hyb-Seq Data: A Case Study of the Balkan Mountain Endemic Cardamine barbaraeoides
Source: Front Plant Sci. 2021 Apr 28;12:659275. doi: 10.3389/fpls.2021.659275 (PMC8115912; doi:10.3389/fpls.2021.659275)
Supplement: Supplementary file 1 [file Data_Sheet_1.zip › Supplementary Table 1.pdf]

Supplementary Table 1. Proportion of homozygous and heterozygous exons recovered in *Cardamine* samples by read-backed phasing of 1,829 targeted exons. In the tetraploid *C. barbaraeoides*, full heterozygosity (i.e. four different alleles obtained per exon) and partial heterozygosity (two to three different alleles per exon) are listed separately. For details on the accession origins, see **Supplementary Data 1**.

Taxon	Population code	Ploidy level	Homozygous	Partially heterozygous			Fully heterozygous
				ratio 1:3	ratio 2:2	ratio 1:1:2	
C. acris subsp. *acris*	C015	2x	54.73%				45.27%
C. acris subsp. *acris*	C019	2x	48.66%				51.34%
C. acris subsp. *pindicola*	C012	2x	65.12%				34.88%
C. acris subsp. *vardousiae*	C004	2x	58.28%				41.72%
C. amara subsp. *balcanica*	C014	2x	58.12%				41.88%
C. amara subsp. *balcanica*	C024	2x	57.19%				42.81%
C. amara subsp. *amara*	C029	2x	53.36%				46.64%
C. amara subsp. *opicii*	C046	2x	79.06%				20.94%
C. apennina	VO	2x	58.99%				41.01%
C. barbaraeoides	C007	4x	10.72%	8.91%	19.96%	46.80%	13.61%
C. barbaraeoides	C010	4x	9.68%	8.31%	23.89%	45.27%	12.85%
C. barbaraeoides	C011	4x	9.68%	7.98%	20.78%	47.51%	14.05%
C. impatiens	C061	2x	89.72%				10.28%
C. lazica	GRA	2x	79.00%				21.00%
C. lazica	TREU	2x	79.99%				20.01%
C. lazica	TRM	2x	80.10%				19.90%
C. matthioli	GRM	2x	55.60%				44.40%
C. penzesii	DEM	2x	67.58%				32.42%
C. rivularis	POB	2x	61.56%				38.44%
C. trifolia	C060	2x	83.38%				16.62%
C. uliginosa	AM1	2x	60.09%				39.91%
C. cf. *uliginosa*	UD	2x	63.26%				36.74%
