# Supplementary material for: Allele Sorting as a Novel Approach to Resolving the Origin of Allotetraploids Using Hyb-Seq Data: A Case Study of the Balkan Mountain Endemic Cardamine barbaraeoides
Source: Front Plant Sci. 2021 Apr 28;12:659275. doi: 10.3389/fpls.2021.659275 (PMC8115912; doi:10.3389/fpls.2021.659275)
Supplement: Supplementary file 1 [file Data_Sheet_1.zip › Supplementary Text 1.pdf]

Supplementary Text 1. Searching for the optimal threshold for unequivocal allele sorting into parental subgenomes

This threshold value is `between_homeolog_distance` variable used in AlleleSorting scripts (https://github.com/MarekSlenker/AlleleSorting). This value is used to decide if the allele pairs identified in an allotetraploid genome are sufficiently different to be attributed to different homeologs (parental subgenomes). If the value is too low, many poorly resolved phylogenetic trees may pass filtering, which can have a negative effect on the final tree estimation. On the other hand, the effect of setting the value too high is in discarding too much data, with no further improvement of the final tree topology. We will demonstrate these effects on the set of 1,829 targeted exons of the tetraploid *Cardamine barbaraeoides*, each one phased to 4 alleles, as used in this study (for the proportions of homozygous, partially or fully heterozygous exons, see **Supplementary Table 1**). We have to emphasize that these effects are data dependent, and have to be explored for each dataset separately.

The following table summarizes the effect of losing data within our dataset caused by the increase in the threshold value. The relative loss of data is visualized in the scatter plot below. The increasing threshold value reaches the plateau effect around 4.

`between_homeolog_distance`	exons passed threshold	exons passed threshold [%]	relative loss of data [%]
0	1829	100	
1	1342.67	73.41	26.59
2	1104.67	60.40	13.01
3	1017.33	55.62	4.77
4	957.67	52.36	3.26
5	905.67	49.52	2.84
6	871.00	47.62	1.90
10	764.67	41.81	1.45
15	678.33	37.09	0.94
20	628.67	34.37	0.54

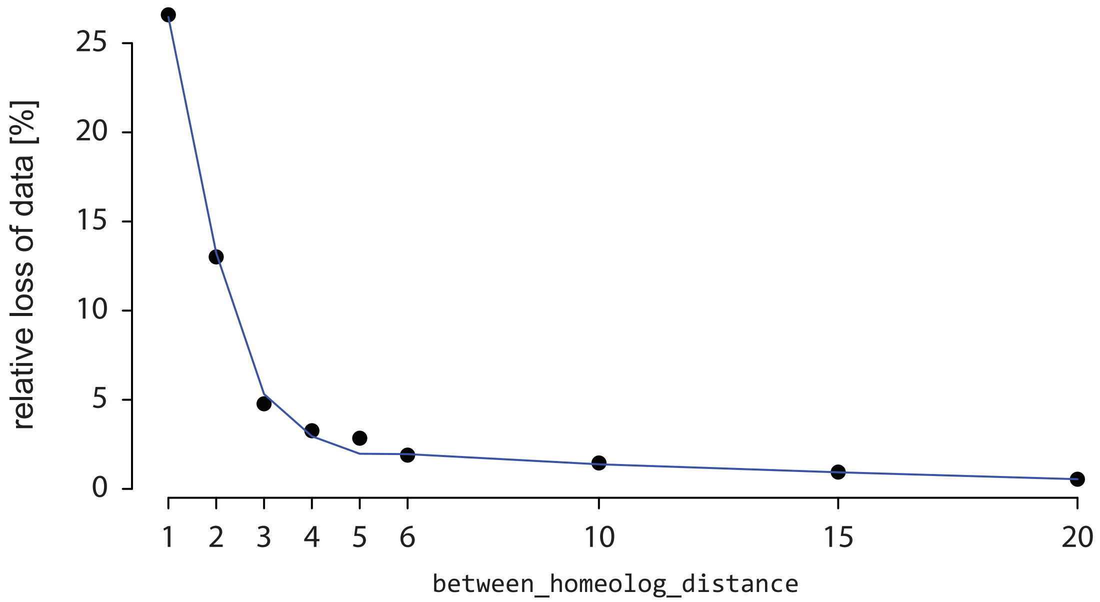

The sequences of *C. barbaraeoides*, which did not pass the given threshold value were removed from the alignment. As a result, we got the alignments of phased sequences of all 1,829 targeted exons of all diploid samples supplemented by 0-3 samples of *C. barbaraeoides*, sorted to ‘A’ and ‘B’ homeologs, for several `between_homeolog_distance` threshold values.

Phylogenetic trees were constructed from each alignment using RAxML-NG. The obtained ML trees were used for species tree inference in ASTRAL-III. The following figure schematically depicts topological changes in the species trees caused by the increasing threshold value.

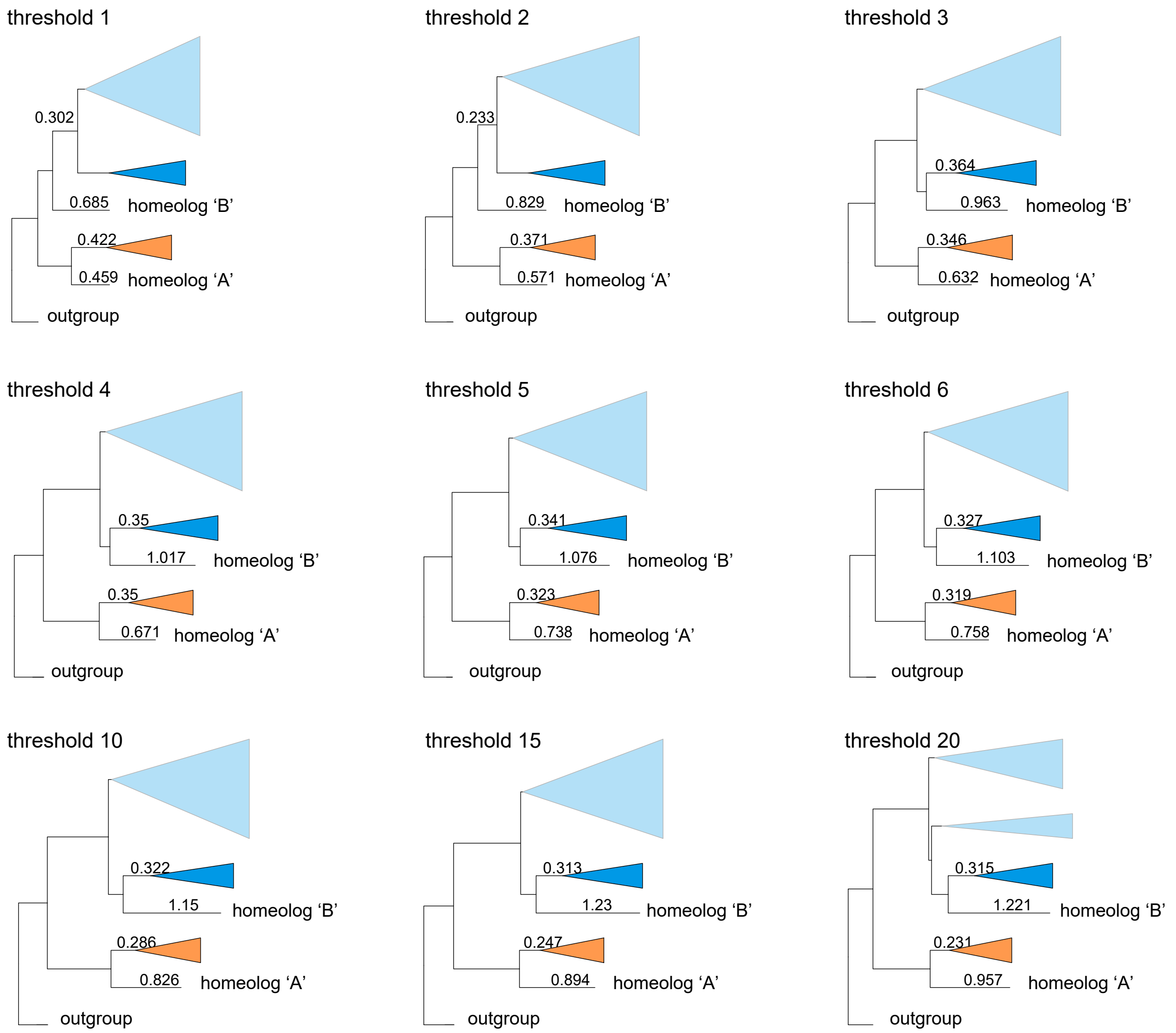

As is obvious from the figure, the positions of ‘A’ and ‘B’ homeologs are changing as the threshold value increases. The ‘A’ homeolog of *C. barbaraeoides* is in a stable sister position to the *C. amara* clade (orange), but the position of ‘B’ homeolog is unresolved when too low threshold values are used. With the increasing threshold value, both homeologs are approaching to the proposed parent clades (orange and blue), but topological changes in favour of one of the parents (within clades) can be excluded.
