# Supplementary material for: Allele Sorting as a Novel Approach to Resolving the Origin of Allotetraploids Using Hyb-Seq Data: A Case Study of the Balkan Mountain Endemic Cardamine barbaraeoides
Source: Front Plant Sci. 2021 Apr 28;12:659275. doi: 10.3389/fpls.2021.659275 (PMC8115912; doi:10.3389/fpls.2021.659275)
Supplement: Supplementary file 1 [file Data_Sheet_1.zip › Supplementary Text 2.pdf]

Supplementary Text 2. Testing if pairs of alleles (sequences) are unequivocally different and attributable to different homeologs.

Alleles obtained from a tetraploid genome are sorted into two distinct homeologs using AlleleSorting scripts (http://github.com/MarekSlenker/AlleleSorting) as follows. First, interallelic distances are computed from the branch lengths of the corresponding ML tree. Distances between alleles are computed as an average length of path connecting all possible allele pairs or trios. As next, we compare the computed distances. If an average distance between alleles within any two pairs is more than `between_homeolog_distance` (the desired threshold)-time shorter than the average distance between alleles within any other possible arrangements, these pairs of alleles are considered to be unequivocally different and attributable to different homeologs. For more details about searching for the optimal threshold value, see **Supplementary Text 1**.

The following table summarizes the formulas used for the calculation of all possible distances among four alleles, and the calculated distances for five examples (trees A-E) of four alleles obtained within one sample.

distance	formula	tree A	tree B	tree C	tree D	tree E
12_34	**average** [(a1_a2) + (a3_a4)]	2	10	14	10	6
13_24	**average** [(a1_a3) + (a2_a4)]	20	20	20	16	8
14_23	**average** [(a1_a4) + (a2_a3)]	20	20	20	16	8
123	**average** [(a1_a2) + (a1_a3) + (a2_a3)]	14	19.33	19.33	14	2.66
124	**average** [(a1_a2) + (a1_a4) + (a2_a4)]	14	19.33	19.33	14	8.66
134	**average** [(a1_a3) + (a1_a4) + (a3_a4)]	14	14	16.66	11.33	8.66
234	**average** [(a2_a3) + (a2_a4) + (a3_a4)]	14	14	16.66	16.66	8.66

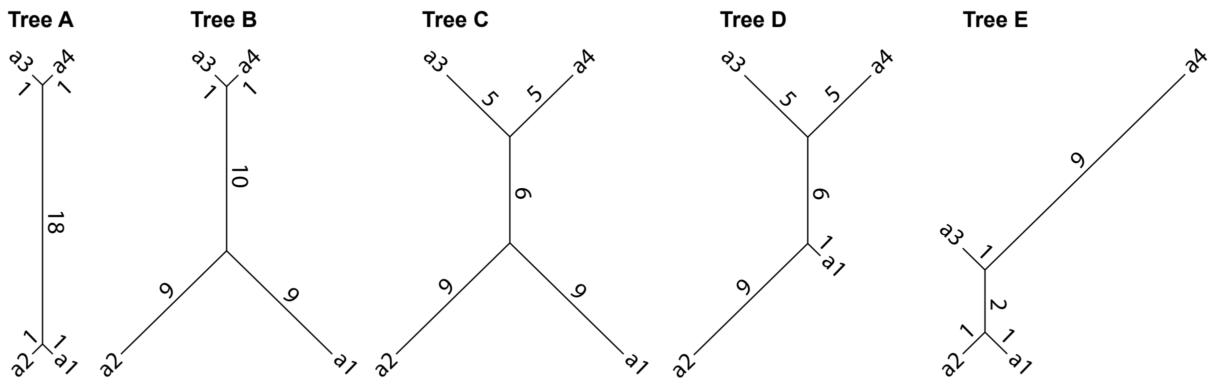

Let's set `between_homeolog_distance = 4`

Tree A: The shortest distance (2) was found in the arrangement as a1 and a2 as one pair and a3 and a4 as another pair. The second shortest distance was found in trios (14). This distance is

more than 4-time longer (`between_homeolog_distance`), thus we consider pairs a1-a2 and a3-a4 as unequivocally different and attributable to different homeologs.

Trees B, C, D: The shortest distance is less than 4-time shorter than the distance of any other possible arrangement. These alleles cannot be unequivocally sorted into homeologs.

Tree E: The shortest distance was found in a trio, what interrupts any further attempts to find two pairs of distinct alleles.
